# Human dendritic cell interactions with the zoonotic parasite *Cryptosporidium parvum* result in activation and maturation

**DOI:** 10.3389/fimmu.2024.1388366

**Published:** 2024-05-10

**Authors:** Ralf Ross, Seyed Sajjad Hasheminasab, Iván Conejeros, Ulrich Gärtner, Faustin Kamena, Andreas Krueger, Anja Taubert, Carlos Hermosilla

**Affiliations:** ^1^ Institute of Molecular Immunology, Justus Liebig University Giessen, Giessen, Germany; ^2^ Institute of Parasitology, Biomedical Research Center Seltersberg (BFS), Justus Liebig University Giessen, Giessen, Germany; ^3^ Institute of Anatomy and Cell Biology, Justus Liebig University Giessen, Giessen, Germany; ^4^ Laboratory for Molecular Parasitology, Department of Microbiology and Parasitology, University of Buea, Buea, Cameroon

**Keywords:** *Cryptosporidium parvum*, dendritic cells, diarrhea, initiation of adaptive immunity, phagocytosis, small intestine

## Abstract

Cryptosporidiosis in humans is caused by infection of the zoonotic apicomplexan parasite *Cryptosporidium parvum*. In 2006, it was included by the World Health Organization (WHO) in the group of the most neglected poverty-related diseases. It is characterized by enteritis accompanied by profuse catarrhalic diarrhea with high morbidity and mortality, especially in children of developing countries under the age of 5 years and in HIV patients. The vulnerability of HIV patients indicates that a robust adaptive immune response is required to successfully fight this parasite. Little is known, however, about the adaptive immune response against *C. parvum*. To have an insight into the early events of the adaptive immune response, we generated primary human dendritic cells (DCs) from monocytes of healthy blood donors and exposed them to *C. parvum* oocysts and sporozoites *in vitro*. DCs are equipped with numerous receptors that detect microbial molecules and alarm signals. If stimulation is strong enough, an essential maturation process turns DCs into unique activators of naïve T cells, a prerequisite of any adaptive immune response. Parasite exposure highly induced the production of the pro-inflammatory cytokines/chemokines interleukin (IL)-6 and IL-8 in DCs. Moreover, antigen-presenting molecules (HLA-DR and CD1a), maturation markers, and costimulatory molecules required for T-cell stimulation (CD83, CD40, and CD86) and adhesion molecules (CD11b and CD58) were all upregulated. In addition, parasite-exposed human DCs showed enhanced cell adherence, increased mobility, and a boosted but time-limited phagocytosis of *C. parvum* oocysts and sporozoites, representing other prerequisites for antigen presentation. Unlike several other microbial stimuli, *C. parvum* exposure rather led to increased oxidative consumption rates (OCRs) than extracellular acidification rates (ECARs) in DCs, indicating that different metabolic pathways were used to provide energy for DC activation. Taken together, *C. parvum*-exposed human DCs showed all hallmarks of successful maturation, enabling them to mount an effective adaptive immune response.

## 1 Introduction

The protozoan parasite *Cryptosporidium parvum* is distributed worldwide and infects both humans and livestock via the fecal-oral route. Neonatal calves represent the main reservoir of this parasite. In healthy adults, it causes severe but self-limiting diarrheal enteritis. Conversely, in immunocompromised individuals, especially HIV-infected persons, newborns, and young children, it can also cause life-threatening chronic cryptosporidiosis. Neither a potent vaccine nor an efficient drug for high-risk patients suffering from cryptosporidiosis is available (1, 2). *C. parvum* is still one of the six major enteropathogens causing severe diarrhea in children younger than 5 years. These facts together with the high prevalence rate of HIV-infected high-risk patients in sub-Saharan Africa and Southeast Asia led the World Health Organization (WHO) in 2006 to include cryptosporidiosis in the group of neglected tropical diseases to draw attention to this disease and encourage research in this field (3). Moreover, neonatal cryptosporidiosis in livestock, affecting mainly calves, lambs, goat kids, and piglets, causes worldwide financial losses among farmers. *C. parvum*-infected mammalian hosts, such as humans and cattle, release billions of infective oocysts that are highly resistant to the environment (4, 5). Infective oocysts can be passed from person to person or from animals to humans (6–8) by direct contact, contaminated drinking water (9), inhaling contaminated dust (10), or eating contaminated food (11). A recent report estimated a global environmental *Cryptosporidium* sp. load from livestock manure of 3.2 × 10^23^ oocysts per year, causing infections on all continents except for Antarctica (12).

Resistant *C. parvum* oocysts, carrying four sporozoites, survive ingestion and low pH of the stomach and release, thereafter, sporozoites within the small intestinal lumen. These sporozoites infect, subsequently, intestinal epithelial cells (IECs). While the *C. parvum* sporozoites enter the IEC cytoplasm, they form a parasitophorous vacuole with a feeder layer, thereby separating the parasite from the host cellular cytoplasm and limiting the exchange between the IEC cytoplasm and the parasite. Thus, *C. parvum* stages (i.e., sporozoites, trophozoites, meronts, gamonts, and oocysts) have an intracellular but extracytoplasmic (=epicellular) localization within the host cell, which may hinder processing and antigen presentation of parasite proteins by the host cell (13). The surface presentation of parasite-derived peptides by MHC class I molecules of IECs is, therefore, probably limited. As *C. parvum* infects only the upper IEC layer from the luminal side, contact with cells of the gut-associated lymphoid tissues (GALTs), residing in deeper layers of the small intestine, is limited. In healthy adults, however, a robust adaptive immune response is elicited and clears the infection. It is well established that dendritic cells (DCs) are mandatory to start any novel adaptive immune response, and their role in activating naïve T cells is summarized in excellent reviews (14, 15). Parasite antigens may be presented to DCs via either M cells or goblet cells. Some studies suggest that apart from infection of the epithelial layer, *C. parvum* might penetrate the epithelium by weakening tight junctions (16) and thereby interacting directly with DCs in mouse models (17, 18). However, only a few danger signals alerting DCs are provided. This may explain the delay of the adaptive immune response that is finally mounted when millions of IECs are infected, thereby releasing enormous numbers of merozoites, gametocytes, and oocysts. At this stage of cryptosporidiosis, danger signals are provided in sufficient amounts to initiate an effective adaptive immune response in immunocompetent patients. This protective immune response intimately involves and depends on cells of the GALTs of the lamina propria, Peyer’s patches, and the mesenteric lymph nodes.

The composition of the GALTs changes markedly during early childhood. Though enteric immunological changes may be essential for effective defense against *C. parvum*, this has not been investigated in detail yet. Several findings on the host innate immunity have shown that polymorphonuclear neutrophils (PMNs), monocytes, macrophages, and IECs are critically involved in protection against this parasite (2, 6, 19–23). After birth, the number of T cells still increases drastically in the intestinal tissues of calves (24); the number of several DC subpopulations rises as well, though at a lower level (24). The relative contribution of DC subpopulations to immunity in the gut, in either humans or cattle, needs further investigation, though considerable progress has been made, especially in humans (25, 26).

As it was not clear yet if *C. parvum* provides pathogen-associated molecular patterns (PAMPs) that are detectable by human DCs or if *C. parvum* employs immune evasion strategies to interfere with DC maturation, we generated human monocyte-derived DCs (MO-DCs) from monocytic blood precursors of healthy blood donors and exposed them to *C. parvum* oocysts and sporozoites *in vitro*. We analyzed key features of DC maturation and detected a robust activation of DCs by *C. parvum*. DCs engulfed parasites by phagocytosis and upregulated activation markers (CD83), antigen-presenting molecules (HLA-DR and CD1a), adhesion molecules (CD11b and CD58), and costimulatory molecules for antigen presentation (CD40 and CD86). New synthesis and surface expression of MHC class II molecules such as HLA-DR ensure that sufficient numbers of parasite-derived peptides can be presented by DCs via MHC class II molecules to naïve T cells. Adhesion molecules stabilize this interaction, and costimulatory molecules provide the necessary signals to overcome the exceptionally high activation barrier of naïve T cells to induce a novel adaptive host immune response (14, 15).

## 2 Materials and methods

### 2.1 *C. parvum* strains and sporozoite excystation

Two *C. parvum* strains, i.e., the commercially available Iowa *C. parvum* strain P102 (Waterborne, New Orleans, USA) and the Leipzig strain (gp60 IIaA15G2RI subtype, University of Leipzig, Germany), were used to investigate the interaction of *C. parvum* with human DCs *in vitro*. The Leipzig strain corresponds to the most commonly reported strain of *C. parvum* in Germany and other developed countries. *C. parvum* was maintained by serial passage of oocysts in 1-day-old calves at the Faculty of Veterinary Medicine of the University of Leipzig, as reported elsewhere (13). Oocysts of the Leipzig strain were isolated from feces of infected calves using a sedimentation/flotation method (27, 28) and maintained at 4°C in sterile phosphate-buffered saline (PBS) (pH 7.4) with penicillin/streptomycin (200 µg/mL; Sigma-Aldrich, Darmstadt, Germany) and amphotericin B (5 µg/mL; Sigma-Aldrich, Darmstadt, Germany). The *C. parvum* oocyst storage medium was exchanged at monthly intervals. Therefore, oocysts were pelleted (5,000 ×*g* for 10 min) and supplemented with fresh medium containing penicillin/streptomycin (13, 27). Before excystation, *C. parvum* oocysts were pelleted at 5,000 ×*g* for 5 min at 4°C to isolate sporozoites (13, 27). Then, acidified (pH 2.0) and sterile pre-warmed (37°C) 1× Hank’s balanced salt solution (HBSS; Sigma-Aldrich, Darmstadt, Germany) was used as oocyst excystation medium (10 min at 37°C). Thereafter, free-released sporozoites were pelleted and incubated at 37°C for 10 min in 1× HBSS with pH 7 (Sigma-Aldrich, Darmstadt, Germany). Once again pelleted, the sporozoites were resuspended in phenol red-free Roswell Park Memorial Institute (RPMI) 1640 cell culture media (Gibco, Darmstadt, Germany) supplemented with 0.3 g/mL l-glutamine, 10% fetal bovine serum (FBS; Gibco, Darmstadt, Germany), and 100 UI penicillin and 0.1 mg streptomycin/mL (both Sigma-Aldrich, Darmstadt, Germany). As the Leipzig strain was no longer available to us, we validated by flow cytometry that the Iowa strain activated MO-DCs to a similar extent. In phagocytosis assays, *bona fide* identical phagocytosis rates were obtained. The effect on DC mobility was comparable as well. 

### 2.2 Generation and cultivation of human monocyte-derived dendritic cells

As a source of immature human DCs, we isolated CD14^+^ monocytes from human blood and generated monocyte-derived dendritic cells (MO-DC) using granulocyte-macrophage colony-stimulating factor (GM-CSF) and interleukin (IL)-4 as described (29). Briefly, buffy coats from healthy donors (*n* = 15) were obtained from the Center of Transfusion Medicine and Hemotherapy of the University Hospital of Giessen and Marburg (UKGM) in Giessen, Germany. The use of buffy coats for research purposes was approved by the local ethics committee of the Justus Liebig University Giessen and UKGM. For the entire isolation procedure and cell culture, exclusively pyrogen-free media, buffers, and equipment were used to exclude unintentional pre-activation and maturation of human DCs; 15 mL aliquots of resuspended buffy coats were diluted 1:1 with DC medium RPMI 1640 (Gibco, Darmstadt, Germany), supplemented with 10% fetal calf serum (FCS) (Sigma-Aldrich, Darmstadt, Germany) and 2 mM l-glutamine and 1 mM sodium pyruvate (both Sigma-Aldrich, Darmstadt, Germany) and overlaid on 15 mL of Biocoll^®^ (Biochrom GmbH, Berlin, Germany) with a density of 1,077 g/mL. Samples were centrifuged for 30 min at 2,500 ×*g* without break. Peripheral blood mononuclear cells (PBMCs) were harvested at the interface of Biocoll^®^ and supernatant, washed several times in MACS^®^ separation buffer with 2% bovine serum albumin (BSA; Miltenyi Biotec GmbH, Bergisch Gladbach, Germany), and subjected to positive selection of CD14^+^ monocytes using immunomagnetic beads and reagents from Miltenyi Biotec GmbH. Briefly, 2.5 × 10^8^ PBMCs and 500 µL Miltenyi human CD14^+^ MicroBeads^®^ were incubated in a total volume of 2.5 mL MACS^®^ buffer for 15 min at 4°C, and CD14^+^ monocytes were isolated using LS Columns and QuadroMACS™ separator according to the recommendations of the manufacturer. A purity of CD14^+^ monocytes of >95% was obtained (data not shown). A total of 3 × 10^6^ monocytes suspended in 3 mL DC medium supplemented with 1,000 U/mL human GM-CSF (ImmunoTools GmbH, Friesoythe, Germany) and 1,000 U/mL human IL-4 (ImmunoTools GmbH) were incubated in 6-well plates (day 0). On day 3, half of the medium was replaced with fresh DC medium with double-concentrated cytokines. Immature MO-DCs were harvested on day 6 of culture and further cultured for the following assays in DC medium either with or without further stimulation. Assays were performed in 24-well plates (Thermo Fisher Scientific, Braunschweig, Germany) in a total volume of 500 µL DC medium with 1 × 10^5^ DCs/well or in 96-well plates (Thermo Fisher Scientific, Braunschweig, Germany) in a total volume of 200 µL with 2 × 10^4^ DCs/well.

### 2.3 Visualization of DC–parasite interaction at ultrastructural level

In order to visualize DC–parasite interactions at ultrastructural level via scanning electron microscopy (SEM), viable *C. parvum* oocysts/sporozoites (ratio 1:3) were co-cultured with human DCs for 120 min on coverslips pre-coated with poly-l-lysine 0.01% (10 mm/diameter; Thermo Fisher Scientific, Braunschweig, Germany). In brief, co-cultures were performed at 37°C and 5% CO_2_ for the entire experimental period. In each well, 2 × 10^5^ DCs and 6 × 10^5^ of the parasite stages were seeded. The cells were then fixed in 2.5% glutaraldehyde (Merck, Darmstadt, Germany) and post-fixed with 1% osmium tetroxide (Merck, Darmstadt, Germany), rinsed in distilled water, dried, CO_2_-treated to the critical point, and lastly sputtered with gold particles. SEM analysis was conducted at the Institute of Anatomy and Cell Biology at the Justus Liebig University Giessen, Germany, utilizing a scanning electron microscope (Philips XL30^®^, Eindhoven, The Netherlands) equipped with a digital camera.

### 2.4 Characterization of DC maturation by flow cytometry

Immature human myeloid DCs from day 6 of culture were harvested, resuspended in fresh DC medium, and exposed to 2 × 10^5^ C*. parvum* oocysts or to lipopolysaccharide (LPS; Sigma-Aldrich, Darmstadt, Germany) or left without further stimulation (24-well plates, total volume of 500 µL DC medium, 1 × 10^5^ DCs/well overnight). For flow cytometry analysis, differently treated DCs were all harvested on day 7 of culture. Aliquots of approximately 5 × 10^5^ DCs were washed in PBS/2% FCS and incubated for 15 min in 10% heat-inactivated human serum to block Fc receptors. After washing, cells were resuspended in sterile 50 µL PBS/2% FCS with 0.5 µg fluorescence-conjugated monoclonal antibodies (mAbs) directed against the CD surface markers CD1a, CD3, CD14, CD19, CD83, and HLA-DR (BioLegend, Amsterdam, Netherlands) or CD11b, CD58, CD40, and CD86 (ImmunoTools GmbH, Friesoythe, Germany) or with isotype control mAbs labeled with the corresponding fluorescence conjugates (BioLegend, Amsterdam, Netherlands). Some samples were fixed and rendered mAb-permeable using FoxP3^®^ fixation (Miltenyi Biotec GmbH, Bergisch Gladbach, Germany) before staining. After incubation for 30 min in the dark, stained cells were washed twice in 1 mL PBS/2% FCS, resuspended after centrifugation in 0.5 mL PBS/2% FCS, and analyzed using a BD Accuri™ C6 flow cytometer (BD Biosciences, San Jose, CA, USA).

### 2.5 Examination of whether human DCs release extracellular traps upon *C. parvum* encounter

To assess potential DC-derived extracellular traps (ETs) after parasite exposure, human DCs on day 6 of culture and *C. parvum* sporozoites/oocysts (DC:*C. parvum* ratio 1:3) were co-cultured [37°C, 5% CO_2_, 120 min, on sterile glass coverslips (15-mm diameter; Thermo Fisher Scientific, Braunschweig, Germany) that had been pre-treated with 0.01% poly-l-lysine (Sigma-Aldrich, Darmstadt, Germany)]. Cell fixation was conducted with 4% paraformaldehyde (Merck, Darmstadt, Germany), and samples were kept at 4°C until final experimentation. To analyze whether DCs are able to release ETs against parasite stages, cells were treated with specific antibodies raised against typical ET components, i.e., anti-myeloperoxidase (MPO) antibodies (1:500; Abcam-ab208670, Darmstadt, Germany) and anti-global histone antibodies (H1, H2A/H2B, H3, and H4; 1:500; Merck, Darmstadt, Germany) [overnight at room temperature (RT)]. Samples were, therefore, subjected to three washes with sterile PBS (Sigma-Aldrich, Darmstadt, Germany) and a 60-min incubation in 2% BSA (Sigma-Aldrich, Darmstadt, Germany) supplemented with 0.3% Triton X100 (Thermo Fisher Scientific, Braunschweig, Germany). Samples were then incubated with primary antibodies overnight followed by incubation in secondary antibody solutions [1:500: IgG #A11005-Alexa 594 goat anti-mouse and IgG #A11008-Alexa 488 goat anti-mouse (Thermo Fisher Scientific, Braunschweig, Germany)] (60 min, RT, total darkness) after three washing steps with sterile PBS (Sigma-Aldrich, Darmstadt, Germany). Anti-fading buffer with DAPI (Thermo Fisher Scientific, Braunschweig, Germany) was used to mount the samples after they had been rinsed three times with sterile PBS. A confocal microscope (Nikon ECLIPSE Ti2, Melville, NY, USA) and an inverted epifluorescence microscope IX81^®^ (Olympus, Tokyo, Japan) equipped with an XM10^®^ digital camera (Olympus, Tokyo, Japan) were both used for analysis.

### 2.6 Release of interleukin-6 and IL-8 of DCs following *C. parvum* encounter analyzed by ELISA

Immature human MO-DCs were harvested on day 6 of culture, resuspended in fresh medium, stimulated with either LPS (Sigma-Aldrich, Darmstadt, Germany) or *C. parvum* stages as indicated, or left without further stimulation. Assays were performed in 96-well plates (Thermo Fisher Scientific, Braunschweig, Germany) in triplicates. Independent ELISAs were performed with MO-DCs generated from three different donors (*n* = 3), yielding comparable results. Commercial ELISA kits were purchased from BioLegend (IL-6) and ImmunoTools (IL-8) and used according to the recommendations of manufacturers. First, capture antibodies were transferred to each well and incubated overnight at RT. After completely removing capture antibodies and washing, a blocking buffer was added and incubated for 1 h at RT. Then, the blocking buffer was removed, and standard and samples were diluted in reagent diluent as indicated and transferred into respective wells. After washing five times with washing buffer, detection antibodies were added, and samples were incubated for 2 h at RT. After washing, poly-HRP-streptavidin (poly-HRP-streptavidin; Hamburg, Germany) was added to each well and incubated for 30 min at RT. After washing, pre-warmed TMB (Sigma-Aldrich, Darmstadt, Germany) was added to each well and incubated for up to 60 min at RT under visual control. Samples were finally analyzed in a Tristar^®^ microplate reader (Berthold Technologies, Bad Wildbad, Germany) at 450 nm.

### 2.7 Phagocytosis of *C. parvum* by DCs

To distinguish between *C. parvum* stages attached to the surface of DCs and phagocytosed parasites, a phagocytosis assay was performed (30), which allows for the discrimination of intra- and extracellular fluorescein-labeled particles by trypan blue quenching. Therefore, *C. parvum* oocysts and sporozoites were labeled with NHS-fluorescein (Sigma-Aldrich, Darmstadt, Germany). *Saccharomyces cerevisiae* specimens were equally processed and used for positive controls as described elsewhere (30). However, in contrast to yeast, *C. parvum* oocysts/sporozoites were not subjected to a 99°C heat-inactivation step and were treated with reduced centrifugation forces to keep them alive. Parts of the *C. parvum* samples were incubated for 30 min at 55°C to kill them before labeling, thereby representing a further control to assess whether sporozoites actively invade human DCs. After visual control of equal fluorescent dye labeling, parasites were added to DCs for 3–4 h to allow phagocytosis. In general, sporozoites and oocysts were distinguishable from one another as well as from DCs by size in FSC/SSC. Moreover, they were detectable in the fluorescence channel FL-1 due to labeling with NHS-fluorescein. After incubation for phagocytosis, cells were harvested and analyzed with and without the addition of trypan blue (Sigma-Aldrich, Darmstadt, Germany). Trypan blue quenches the green fluorescence of free parasites but not of phagocytosed ones, as living cells remain negative for trypan blue, and direct contact with trypan blue is needed for quenching. Green fluorescence was detected in FL-1, while red fluorescence due to quenching was detected in FL-4. As a further control, DC-mediated phagocytosis was blocked with cytochalasin D (10 µg/mL; Merck, Darmstadt, Germany).

### 2.8 Migratory activity of DCs exposed to *C. parvum* via live cell 3D holotomographic microscopy analysis

Human DCs on day 6 of culture were either subjected to phagocytosis without pre-stimulation or in some experiments incubated with LPS overnight before *C. parvum* oocysts/sporozoites were added on day 7. In total, samples were monitored for 3–5 h by fluorescence microscopy. Therefore, DCs were resuspended in an imaging medium including 20 μM Hoechst (Thermo Fisher Scientific, Braunschweig, Germany) and 0.1% BSA (Sigma-Aldrich, Darmstadt, Germany). Sterile ibidi^®^ plastic cell culture plates (dish 35 mm^2^ with low profile, ibidi^®^, Gräfelfing, Germany) were used to seed 1 mL of this cell solution (0.5 × 10^6^ DCs) before incubation at 5% CO_2_ and 37°C in an incubation cell chamber (ibidi^®^ dish 35 mm^2^). For DC settling and to prevent condensation on the ibidi^®^ incubation chamber lid, a 30-min resting period at 37°C was employed. Subsequently, 2 × 10^5^ NHS-fluorescein-labeled oocysts/sporozoites were added into the chamber and incubated for 3–5 h. Using a Fluo-3D Cell Explorer^®^ (Nanolive, Lausanne, Switzerland) equipped with a ×60 magnification (λ = 520 nm, sample exposure 0.2 mW/mm^2^) and a field depth of 30 µm, the samples were analyzed. Image capture was configured for refractive index (RI) and Hoechst (blue channel) detection (Thermo Fisher Scientific, Braunschweig, Germany) acquiring images every minute for 3–5 h. Thereafter, each channel was independently exported using Steve^®^ software v.2.6 (Nanolive, Lausanne, Switzerland) and processed using Image J^®^ software (Fiji version 1.7; NIH, USA). Using RI values from the images, digital staining was accomplished thereafter to achieve 3D holotomographic imaging.

### 2.9 *C. parvum* enhances oxygen consumption rates of human DCs

To estimate glycolytic and oxidative responses, ECAR and OCR values of human MO-DCs were quantified using the metabolic extracellular flux analyzer Seahorse XFp^®^ (Agilent, Ratingen, Germany) according to the recommendations of the manufacturer (31); 1 × 10^5^ cells in 50 µL were carefully transferred into each well of an 8-well XF^®^ Seahorse analyzer plate (Agilent, Ratingen, Germany), which had been coated previously with 0.001% poly-l-lysine for 30 min (Sigma-Aldrich, Darmstadt, Germany). Then, 50 μL of XF^®^ assay medium (Agilent, Ratingen, Germany) was administered to control wells. Subsequent to a 45-min incubation period at 37°C without CO_2_ supplementation, 130 µL of XF^®^ assay medium was supplemented to each well to achieve a final volume of 180 µL per well. After 6 cycles of baseline measurement in the Seahorse XF^®^, a suspension of *C. parvum* (3 × 10^5^ oocysts/20 μL) or 20 µL aliquots containing other microbial components or pure XF^®^ assay medium were added via instrument-own injection ports, and ECAR/OCR values were continuously detected for additional 30 cycles. OCR and ECAR registries were evaluated using Wave^®^ software (Desktop Version, Agilent, Ratingen, Germany) as previously stated elsewhere (31, 32).

## 3 Results

### 3.1 Exposure to *C. parvum* stages induces cluster formation and enhances surface expression of adhesion molecules CD11b and CD58 of MO-DCs

For this study, human MO-DCs were generated in sufficient purity and viability in an immature state to allow for subsequent maturation by co-culture with *C. parvum*. To this end, pyrogen-free disposable equipment and media were employed for the entire isolation procedure and cell culture to exclude unintentional pre-activation and maturation of the MO-DCs. CD14^+^ monocytes were enriched from PBMCs by positive selection with paramagnetic beads attached to anti-CD14 antibodies. CD14 is part of the LPS receptor of monocytes and macrophages but cannot signal by itself, as it has no intracellular domain. The purity of positive selected CD14^+^ monocytes is higher, fewer particles like thrombocytes are included, which may interfere, and truly immature DCs can be generated as frequently and reliably as shown in numerous publications including our own (28) and this study. As shown in **Supplementary Figure 1A**, the purity of CD14^+^ monocytes was >95% in the positive fraction. MO-DCs harvested on day 6 of culture following cultivation with GM-CSF and IL-4 by gentle rinsing of the non-adherent MO-DCs again yielded purity > 95% by flow cytometry. Stimulation with LPS indicated upregulation of all activation markers, as expected (**Supplementary Figure 1B**). The viability of monocytes and MO-DCs as indicated by trypan blue staining was >95% as well. If not mentioned otherwise in the text, all immature DCs were harvested on day 6 of culture, incubated in fresh medium with or without *C. parvum* or other stimuli, and analyzed depending on the assay either the same day or on day 7. Regardless of stimulation, all samples of the same assay were incubated under identical conditions for exactly the same time.

**Figure 1:**
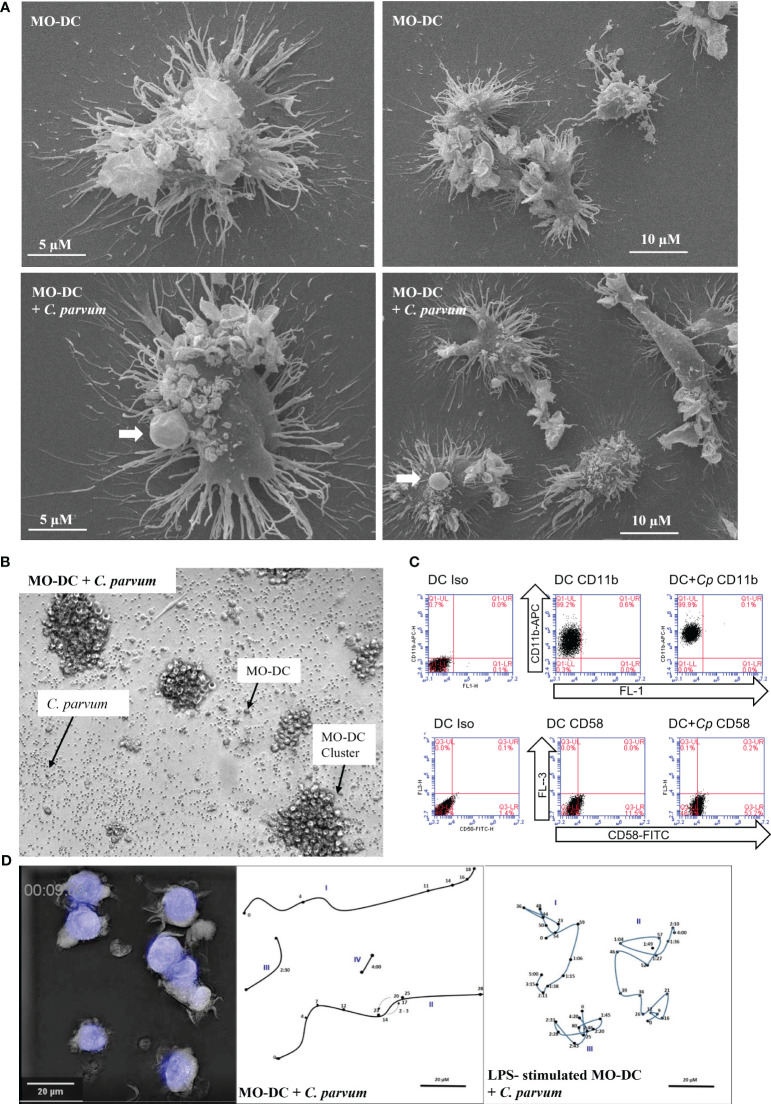
Monocyte-derived dendritic cells (MO-DCs) show signs of maturation following co-culture with *Cryptosporidium parvum*. **(A)** Interaction of DCs with *C*. *parvum* was examined using scanning electron microscopy (SEM). Single DCs (left panels) and overviews with lower magnification are displayed (right panels). Exposure of human DCs to *C*. *parvum* oocysts for 120 min (lower panels) does not interfere with the typical elaborated structure of DCs (upper panels). *C*. *parvum* oocysts attached to DCs (lower panels) are indicated by arrows. **(B)** Aggregation of MO-DCs following activation with *C*. *parvum*. Single oocysts, MO-DCs, and DC clusters are indicated by arrows. **(C)** Adhesion molecules CD11b and CD58 were upregulated after stimulation of DCs with *C*. *parvum.* Percentage of positive cells in the respective quadrant is as follows: CD11b; DC, 99%; DC + Cp, 100%; nearly all cells are positive mean fluorescence rises from 51,975 (DC) to 137,910 (DC + Cp); CD54; DC, 12%; DC + Cp, 54%. Data are representative of *n* = 3 experiments with cells from different donors. **(D)** 3D holotomographic live cell imaging of human DCs with *C*. *parvum*. An outtake showing morphology of DCs and *C*. *parvum* oocysts is depicted (left panel). Movement of single DCs (Roman numbers) was traced (middle and right panels). Arabic numbers indicate positions after corresponding time in minutes/hours. Human DCs were either co-incubated with *C*. *parvum* directly (middle panel) or following overnight stimulation with lipopolysaccharide (LPS) (right panel). Videos are available in the **Supplementary Material**.

The elaborated structure of DCs is of functional relevance. Veiled structures are involved in migration and interaction with antigens (see **Supplementary Videos**), and ultrathin dendrites enlarge the surface and enhance the chance to interact with naïve T cells, detecting fitting peptides in context with MHC molecules in the T-cell areas of the draining lymph nodes. The SEM-based ultrastructural analysis identified both types of dendrites upon MO-DCs cultured alone or co-cultivated with *C. parvum* oocysts. MO-DCs are morphologically indistinguishable from non-exposed control cells (**Figure 1A**). *C. parvum* oocysts interacting with DCs are indicated by arrows.

As sentinel cells of the immune system, DCs express an extraordinarily high number of pattern recognition receptors (PRRs) to identify invasive pathogens (14, 15) including prototypic molecules of major groups of microorganisms like yeasts and gram-positive or gram-negative bacteria. Work on human cell lines and mouse models hint at a potential role of TLR-3 or TLR-4 in the detection of *C. parvum* (33, 34), but even immune-suppressive mechanisms of *C. parvum* could not be ruled out. Thus, we used the gold standard TLR-4 ligand LPS and *C. parvum* oocysts either alone or in combination in the first experiment to stimulate MO-DCs and analyzed the effect on the expression of HLA-DR antigen-presentation molecules on MO-DCs. Both stimuli either alone or in combination enhanced the surface expression of HLA-DR (**Supplementary Figure 2A**), indicating maturation of DCs and not inhibition.

*C. parvum*-driven stimulation of MO-DCs was also apparent microscopically by boosted cluster formation (**Figure 1B** and **Supplementary Figure 2B**). During the generation of DCs from monocytes by culture with GM-CSF and IL-4, cells typically build dendrites and form clusters of cells. When DCs are harvested, these aggregates disintegrate due to resuspension. Without further stimulation, DCs then remain more or less evenly distributed in the wells. In current experiments, both LPS stimulation and *C. parvum* exposure equally induced significant cluster formation of MO-DCs (**Figure 1B**, **Supplementary Figure 2B**). In **Figure 1B**, single oocysts, single MO-DCs, and MO-DC clusters are illustrated and indicated by arrows. Strikingly, a concentration as low as 2.5 pg/mL of LPS was sufficient to induce DC aggregation (**Supplementary Figure 1A**), indicating that human DCs effectively responded to such trace amounts of microbial substances with maturation. Moreover, DC aggregation after *C. parvum* exposure was direct proof of parasite-driven MO-DC movements, interactions, and activation. Consistent with this finding, we found that parasite exposure led to an enhanced expression of the adhesion molecules CD58 (LFA-3) and CD11b (integrin α-M) on the surface of MO-DCs (**Figure 1C**). An increase of adhesion molecules is required to improve interaction with T cells *in vivo*, and clustering indicates active movement as required *in vivo* to move to the place of infection to take up antigens, then to the lymph vessels, and finally to the T-cell areas in the lymph nodes.

### 3.2 3D holotomographic live cell imaging of human DCs with *C. parvum*

Additionally, we used live cell 3D holotomographic microscopy (3D Cell Explorer^®^, Nanolive, Lausanne, Switzerland) to better illustrate the early effects of *C. parvum* oocyst/sporozoite confrontation on MO-DC shape and motility. Overall, migratory activities of DCs toward *C. parvum* were monitored either with or without LPS pre-stimulation overnight on day 7 of culture. As visible in Videos 1 and 2 (see the **Supplementary Material**), several DCs were highly motile also in the case of double stimulation (*C. parvum* + LPS), indicating that motility was not suppressed by *C. parvum* contact. While DCs moved over comparable distances with or without LPS pre-stimulation, LPS-treated cells seemed more adherent and changed direction more frequently, while cells without LPS pre-stimulation moved straight on for longer distances. The morphology of DCs and tracks of selected cells with calculated distances covered per time are depicted (**Figure 1D**).

### 3.3 *C. parvum* exposure drives MO-DC maturation by upregulating selected antigen presentation-associated and costimulatory DC markers

**Figure 2:**
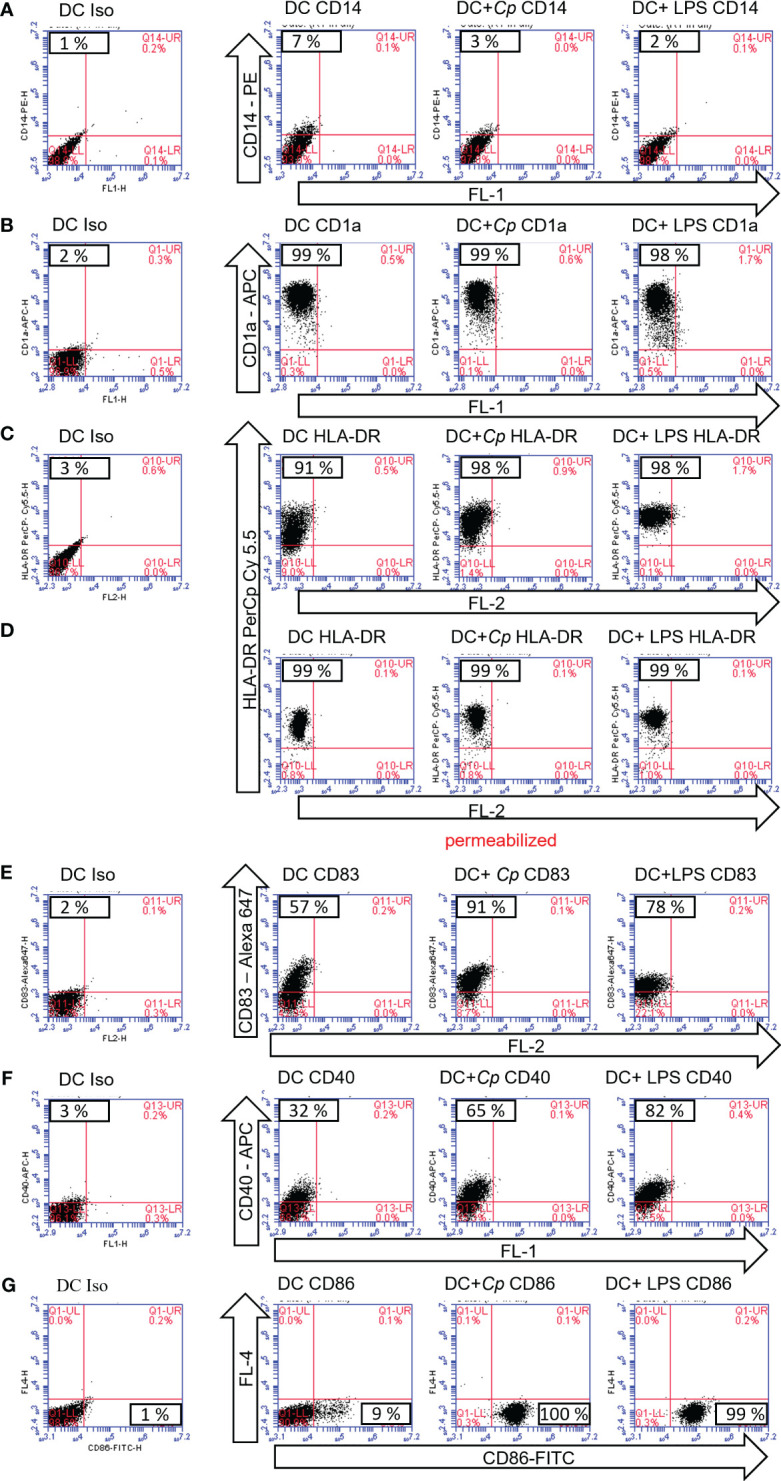
Maturation markers of monocyte-derived dendritic cells (MO-DCs) are upregulated following exposure to *Cryptosporidium parvum*. Flow cytometry of MO-DCs incubated on day 6 of culture with or without stimulation with *C. parvum* oocysts (*Cp*) or lipopolysaccharide (LPS) and analyzed on day 7. Cell surface markers were detected with fluorescence-labeled monoclonal antibodies. Target molecules and conjugates are indicated (arrows). Unused fluorescence channels are indicated and named by channels FL-1–4 (arrows). Samples in **(D)** were perforated to allow for intracellular HLA-DR staining. Percentage of positive cells is depicted in the respective quadrants of the subfigures, i.e., the upper left quadrant **(A–F)** or the lower right quadrant **(G)**.

A typical activation-driven expression pattern was observed following stimulation of immature MO-DCs on day 6 with or without *C. parvum* stages or LPS overnight followed by analysis on day 7 of culture. Unstimulated and stimulated DCs had already downregulated CD14 (**Figure 2A**). The antigen-presenting molecule CD1a, which allows DCs to present microbial lipid antigens to T cells, was already upregulated (**Figure 2B**). The latter marker indicated that the cells were healthy and survived quantitatively during cultivation. Human HLA-DR molecules are required to present microbial peptides to (naïve) T cells. Since exclusively professional antigen-presenting cells (APCs) express MHC class II molecules while nearly all cells express MHC class I molecules, human MHC class II (HLA-DR) was selected for the current analysis. As expected, HLA-DR was already expressed on isolated monocytes and enhanced on day 6 of culture (not shown). This molecule was further upregulated on day 7 by both LPS and *C. parvum* stimulation (**Figure 2C**). However, the expression levels of HLA-DR were quite heterogeneous, which may be based on a high number of molecules still being located on intracellular membranes or within intracellular vesicles and not yet loaded with peptides. Therefore, permeabilized MO-DCs were additionally tested under identical culture conditions. Indeed, permeabilization resulted in more focused staining, thereby confirming parasite-driven HLA-DR upregulation (**Figure 2D**). Of note, both processes, i.e., increased synthesis and peptide loading of new HLA molecules and peptide loading of intracellularly stored but unloaded HLA molecules at the time point of pathogen exposure, increase the likelihood that sufficient numbers of microbial peptides are presented among a vast majority of irrelevant peptides. This process is essential to induce a proper adaptive immune response against pathogenic microorganisms. Obviously, in the case of *C. parvum* stage exposure, these reactions were efficiently driven, finally resulting in MO-DC activation and maturation.

Moreover, CD83, a reliable frequently used maturation marker of human DCs, was upregulated upon stimulation with either LPS or *C. parvum* (**Figure 2E**). The same was true for the costimulatory molecules CD40 (**Figure 2F**) and CD86 (**Figure 2G**) when compared to unstimulated control cells. Together with CD80, they represent the most important costimulatory molecules of DCs, which are especially needed at high densities to successfully stimulate naïve T cells. In contrast to CD40 and CD86, CD80 was already highly expressed on immature DCs (data not shown). Taken together, all markers displayed indicated the maturation of MO-DCs upon parasite exposure (**Figure 2**). The data are representative of three independent experiments using DCs generated from different blood donors (*n* = 3).

### 3.4 Exposure to *C. parvum* stages triggers significant IL-6 and IL-8 secretion by MO-DCs

**Figure 3:**
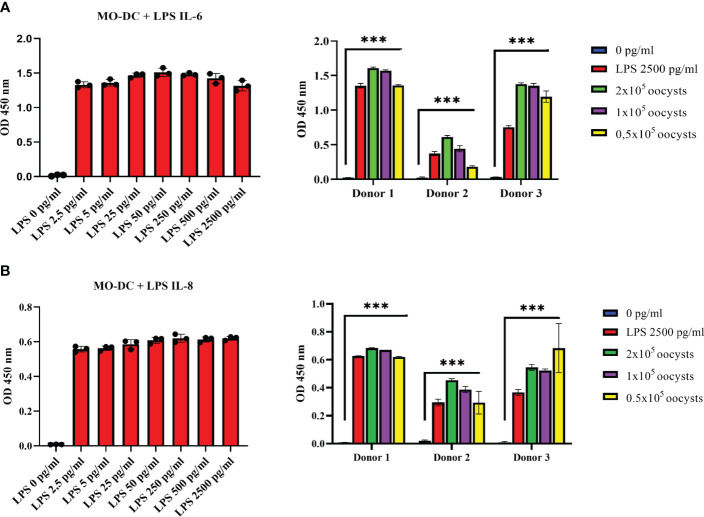
Monocyte-derived dendritic cells (MO-DCs) secrete the pro-inflammatory cytokines IL-6 and IL-8 following co-culture with *Cryptosporidium parvum*. Human immature MO-DCs generated from CD14+ monocytes with granulocyte-macrophage colony-stimulating factor (GM-CSF) and IL-4 were stimulated on day 6 of culture either with increasing concentrations of lipopolysaccharide (LPS) (left panels) or with *C*. *parvum* oocysts or 2500 pg/mL LPS (right panels). Human IL-6 **(A)** or IL-8 **(B)** in the supernatant was detected by ELISA. LPS titration (left panels) is representative of *n* = 5 independent experiments. Data on the right panels were obtained from three different blood donors in independent experiments. Mean values of triplicates are displayed. Either by stimulation with *C*. *parvum* oocysts or LPS, respectively, IL-6 and IL-8 levels in the cell supernatants increased highly significantly (***p ≤ 0.0001) compared with unstimulated DCs (right panels).

DCs secrete a number of cytokines and chemokines to induce and regulate adaptive immune responses (15). In the current study, MO-DCs were exposed to *C. parvum* stages, and IL-6- and IL-8-based responses were quantified. Overall, IL-6 is an important molecule for the induction of Th-1 cells, Th-17 cells, and regulatory T cells. IL-8 represents a chemokine that is relevant for attracting other effector cells of the innate immune system like PMNs, which were already shown to hamper *C. parvum* host cell invasion by phagocytosis or NETosis (35, 36). In preliminary assays, the functionality of the tests was confirmed using LPS stimulation as positive controls (**Figure 3**, left panels). While the expression level of both factors was low without stimulation, trace amounts of LPS (2.5 pg/mL) were sufficient to yield almost maximal secretion levels. Thus, the culture supernatants had to be diluted fivefold (IL-6) or 10-fold (IL-8) compared to the recommendations of the manufacturers to avoid exceeding the upper detection limit. Overall, LPS-induced IL-6 and IL-8 secretion was induced at least 30- and 70-fold, respectively (**Figure 3**, right panels). Referring to *C. parvum* exposure, MO-DCs responded with high significance (*C. parvum* vs. control: *p* ≤ 0.0001) with IL-6 (**Figure 3A**) and IL-8 secretion (**Figure 3B**). When stimulating MO-DCs with different numbers of parasites, a slight dose dependency of cytokine production was apparent. The different donors showed typical individual quantitative variations in their responses, but in principle, all reacted by strong cytokine production. Of note, the strength of parasite-driven reactions was considerably high since the total cytokine/chemokine levels upon *C. parvum* stimulation following dilution of the supernatants even exceeded cytokine levels obtained from LPS-stimulated DCs and as well the highest values of the standard curve, so the cytokine concentration of IL-6 and IL-8 is not displayed in the figure. *C. parvum* stimulation induced, however, several thousand pg/mL of IL-6 and IL-8, while unstimulated cells expressed only less than 10 pg/mL of these cytokines. Moreover, oocysts and excystation material from the same number of oocysts induced high levels of IL-6 and IL-8 secretion, indicating that protein suspensions from *C. parvum* were sufficient for human MO-DC activation (**Supplementary Figure 3**).

### 3.5 *C. parvum* stages do not induce extracellular trap formation in human MO-DCs

*C. parvum* oocyst and sporozoite stages are reported to induce NET formation in human and bovine PMNs (33), and a recent publication suggested ET formation by CD123^+^ plasmacytoid DCs (37). Here, we studied, therefore, whether human MO-DCs may perform ETosis upon exposure to *C. parvum* stages. Overall, both DNA and histones were exclusively detected in the nuclear compartment of MPO-labeled DCs. Stimulation with neither *C. parvum* nor PMA induced ET formation in human MO-DCs (**Supplementary Figure 4**).

### 3.6 Human MO-DCs efficiently phagocytize *C. parvum* oocysts and sporozoites

In general, a robust immune response is driven when DCs directly phagocytize microorganisms. DCs may phagocytize any particles <1–5 µm, but uptake efficiency is markedly enhanced if DCs sense microbial antigens by PAMP receptors or based on complement/antibody-mediated opsonization. As phagocytosis is a fundamental and efficient mechanism of the human immune defense, we here intended to assess DC phagocytic capacities upon *C. parvum* confrontation (38, 39).

**Figure 4:**
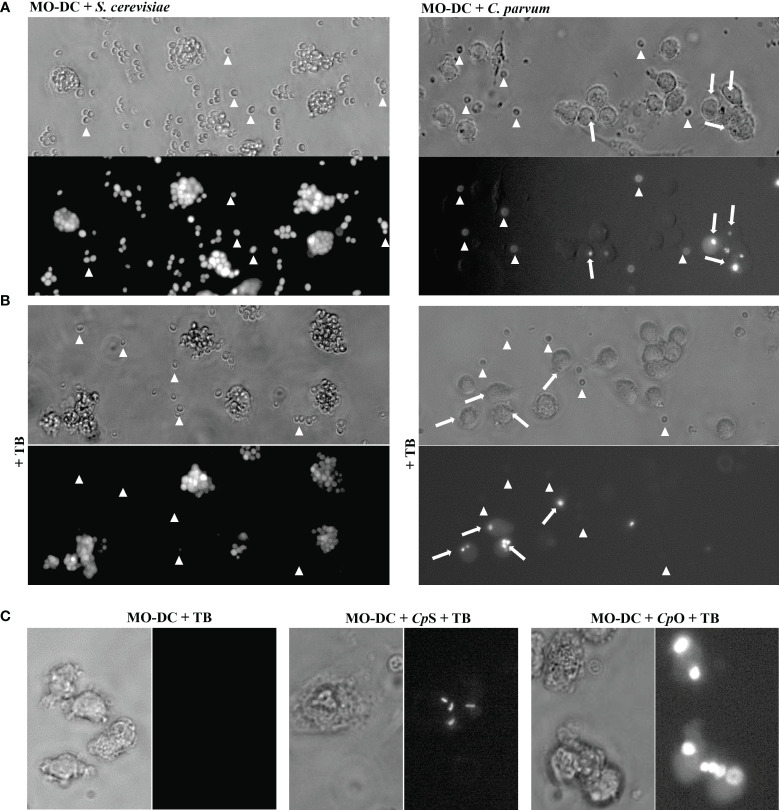
Monocyte-derived dendritic cells (MO-DCs) take up *Cryptosporidium parvum* oocysts and sporozoites as indicated by fluorescence microscopy. MO-DCs on day 6 of culture were either left untreated or stimulated with *C*. *parvum* oocysts (CpO), *C*. *parvum* sporozoites (CpS), or yeast (*Saccharomyces cerevisiae*) for 3 (h) Brightfield and fluorescence views of the same area are depicted next to each other. Trypan blue (TB) was added to the samples just before microscopy, as indicated. **(A)** MO-DCs incubated with yeast (*S. cerevisiae*; left panel) or *C*. *parvum* oocysts (right panel) were analyzed. Extracellular fluorescent microbes are indicated by arrowheads and intracellular phagocytosed microbes by arrows. Extracellular microbes are clearly detectable in fluorescence pictures as well. **(B)** Like panel A but with TB in the medium. Intracellular microbes are still clearly detectable by fluorescence but not extracellular microbes. Please note that MO-DCs phagocytosed large clusters of *S. cerevisiae* but only few *C*. *parvum* oocysts (arrows). **(C)** DCs treated with TB with intracellular fluorescent *C*. *parvum* oocysts (CpO) and sporozoites (CpS) are clearly detectable, while DCs alone are not detectable.

**Figure 5:**
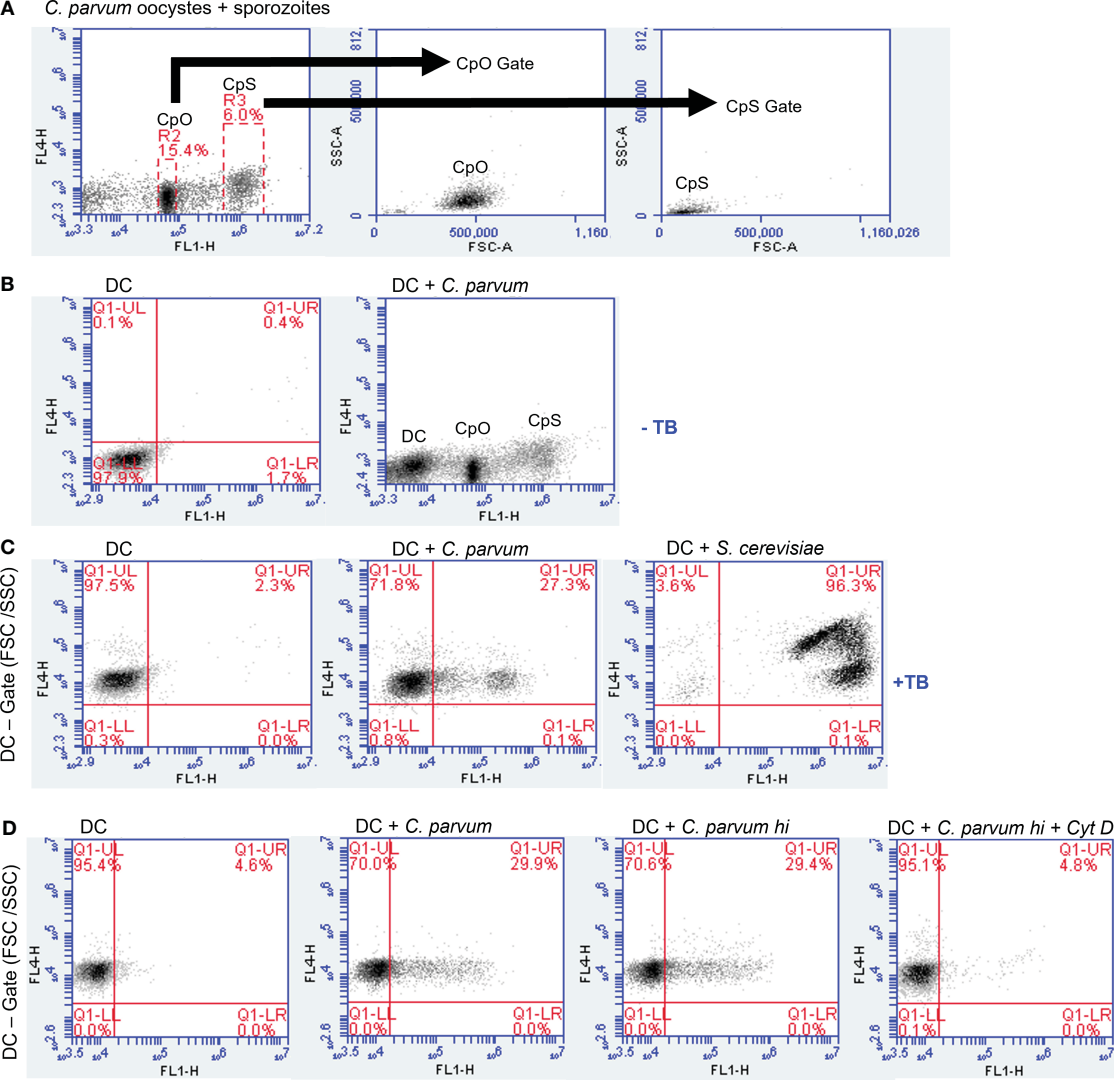
Monocyte-derived dendritic cells (MO-DCs) take up *Cryptosporidium parvum* as indicated by flow cytometry. MO-DCs on day 6 of culture were either left untreated or stimulated with *C*. *parvum* oocysts (CpO) and sporozoites (CpS) or yeast (*Saccharomyces cerevisiae*) and analyzed by flow cytometry. **(A)** A mixture of *C*. *parvum* oocysts and sporozoites was labeled as described. Two distinct populations of green fluorescent particles were detectable (left panel). Gating revealed in FSC/SSC that the stronger fluorescence marks the smaller sporozoites. **(B)** MO-DCs are less fluorescent (left panel) and form a distinct population in channel FL-1 (right panel). **(C)** Addition of trypan blue to the medium (TB) induces a red fluorescence of DCs in channel FL-4. Three hours after incubation with *C*. *parvum* (middle panel) and *S. cerevisiae* (right panel), a green fluorescent population is detectable in FL-1, while an FSC/SSC gate excludes smaller particles than DCs. Trypan blue labeling quenches green fluorescence of externally adherent particles. Note that approximately 25% of DCs have phagocytosed *C*. *parvum*, while nearly all DCs have phagocytosed *S. cerevisiae*. **(D)** Heat killing of *C*. *parvum* has no effect on phagocytosis (hi; third panel), while cytochalasin D (Cyt D) abrogates phagocytosis.

Light microscopy implicated that *C. parvum* oocysts were efficiently phagocytized by activated human DCs (not shown). For direct proof of phagocytosis and quantification, we applied a phagocytosis assay (29), which allows the discrimination between intracellular and extracellular fluorescein-labeled particles. For positive controls, fluorescein-labeled *S. cerevisiae* was included in the experiments. A mixture of fluorescence-labeled *C. parvum* sporozoites and oocysts was used to expose MO-DCs on day 6 of culture (**Figures 4A**, **5A**). When assessing parasite fractions, flow cytometry analysis unveiled two distinct populations in FL-1/FL-4 with fluorescence in FL-1 (gates R2 and R3). Particles with higher FL-1 fluorescence (gate R3) were smaller than particles from gate R2 as shown in FSC/SSC plots, indicating that *C. parvum* sporozoites were labeled with higher fluorescence intensity than oocysts (**Figure 5B**). This was in line with fluorescence microscopic images of sporozoites/oocysts. However, a small proportion (approximately 10%–15%) of oocysts displayed a higher fluorescence intensity than other oocysts in microscopy (**Supplementary Figure 5**). They may contribute to the signals between gates R2 and R3 and may represent the less abundant thin-walled type of oocysts. To distinguish between DC-adherent and DC-internalized parasites, trypan blue was added to the medium and adjusted to a pH of 4.0 using a citrate buffer just before analysis. Consequently, the green fluorescence of external parasites was quenched (**Figure 4B**, arrowheads). Intracellular parasites, in contrast, were protected from trypan blue staining and, therefore, still displayed a green fluorescence (**Figure 4B**, arrows). Uptake of either *C. parvum* oocysts or sporozoites was clearly detectable (**Figure 4C**). The general gating strategy is depicted in **Figures 5A, B**. In **Figure 5C**, cells were treated with trypan blue, and DCs were gated by size in FSC/SSC. Consequently, all cells are in the upper left quadrant because DCs were not labeled (left panel). Some DCs previously exposed to fluorescein-labeled *C. parvum* (middle panel) or *S. cerevisiae* (right panel) for 3–4 h, however, displayed a green fluorescence (FL-1, upper right) due to phagocytized microbes.

Overall, the current data indicated that ~25% of DCs indeed phagocytized *C. parvum* stages (**Figure 5C**). In contrast, nearly all DCs took up *S. cerevisiae* (96.3%, **Figure 5C**) during the same time period, though *S. cerevisiae* is bigger and uptake is more difficult. Maybe DCs were activated more efficiently by *S. cerevisiae* based on yeast-derived PAMPs (e.g., TLR-2-ligand zymosan). The phagocytosis inhibitor cytochalasin D blocked the uptake of *C. parvum*, while heat killing of *C. parvum* (55°C for 30 min) did not influence this effector mechanism, indicating that uptake was truly by DC-derived phagocytosis and not by sporozoite invasion (**Figure 5D**).

### 3.7 Exposure to *C. parvum* stages induces oxidative responses in human MO-DCs

**Figure 6:**
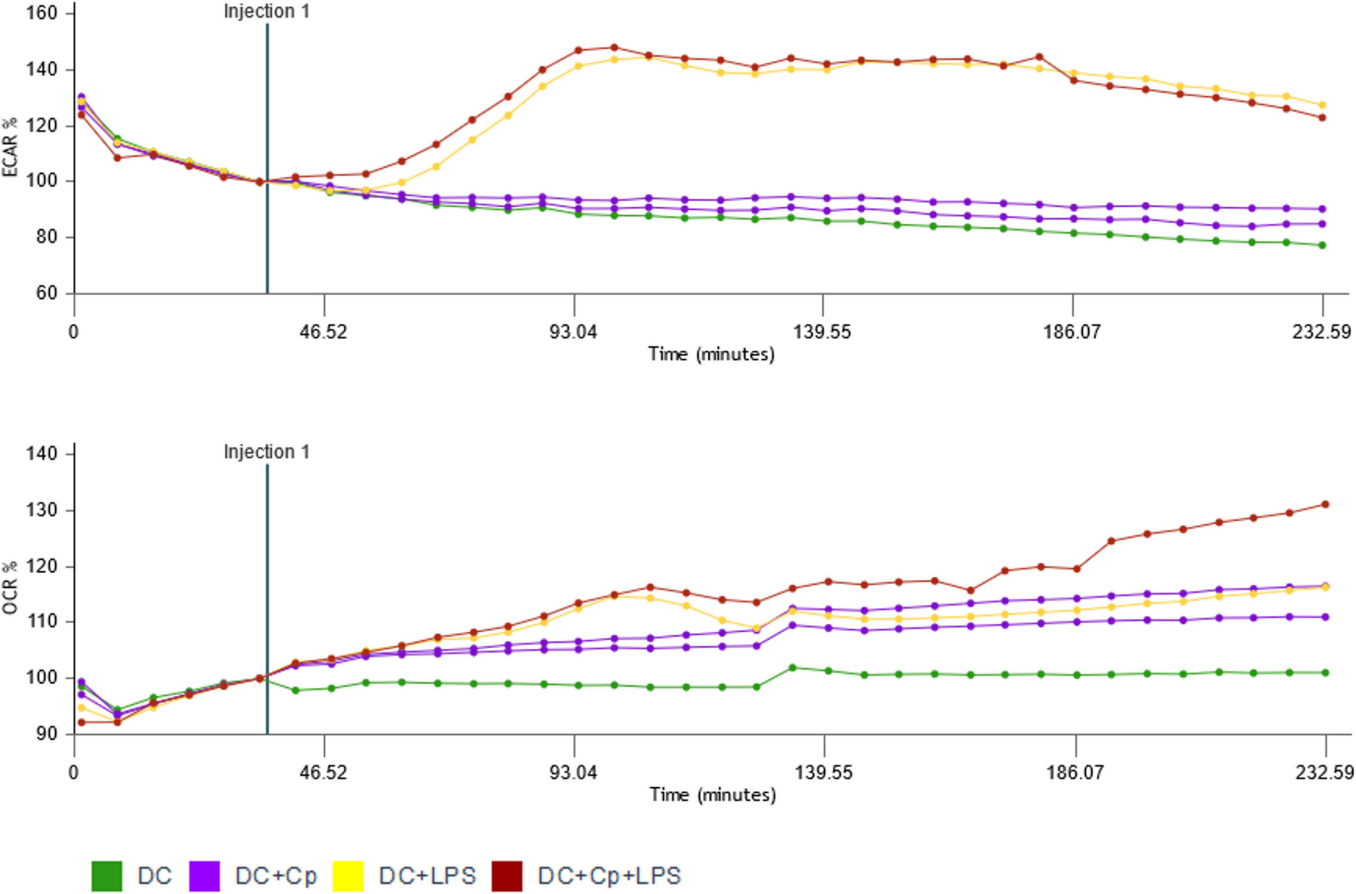
Monocyte-derived dendritic cells (MO-DCs) take up *Cryptosporidium parvum* oocysts and sporozoites as indicated by fluorescence microscopy. MO-DCs on day 6 of culture were either left untreated or stimulated with *C*. *parvum* oocysts (CpO), *C*. *parvum* sporozoites (CpS), or yeast (*Saccharomyces cerevisiae*) for 3 (h) Brightfield and fluorescence views of the same area are depicted next to each other. Trypan blue (TB) was added to the samples just before microscopy, as indicated. **(A)** MO-DCs incubated with yeast (*S. cerevisiae*; left panel) or *C*. *parvum* oocysts (right panel) were analyzed. Extracellular fluorescent microbes are indicated by arrowheads and intracellular phagocytosed microbes by arrows. Extracellular microbes are clearly detectable in fluorescence pictures as well. **(B)** Like panel A but with TB in the medium. Intracellular microbes are still clearly detectable by fluorescence but not extracellular microbes. Please note that MO-DCs phagocytosed large clusters of *S. cerevisiae* but only few *C*. *parvum* oocysts (arrows). **(C)** DCs treated with TB with intracellular fluorescent *C*. *parvum* oocysts (CpO) and sporozoites (CpS) are clearly detectable, while DCs alone are not detectable.

Energy for human DC maturation is provided by different metabolic pathways including glycolysis, detectable by an increase in ECAR, and/or oxidative phosphorylation, detectable by an increase in OCR. Stimulation of immature MO-DCs on day 6 of culture with different stimuli like LPS, *S. cerevisiae*, flagellin, or PMA + ionomycin all increased ECAR, thereby indicating enhanced glycolytic responses (**Supplementary Figure 6**). In contrast, exposure of MO-DCs to *C. parvum* stages failed to induce glycolytic responses (**Figure 6**). However, the combination of LPS and *C. parvum* triggered equal glycolytic responses, and LPS alone denied any inhibitory effect of the parasite. In contrast to ECAR, oxidative responses of MO-DCs were indeed induced by *C. parvum* stages since OCR slowly but steadily increased following parasite exposure, to a similar extent as for LPS stimulation. In line with this, double stimulation with *C. parvum* + LPS showed additive effects on OCR values (**Figure 6**).

## 4 Discussion

We show that *C. parvum* stages induce full maturation of human MO-DCs. All aspects tested are required to turn DCs into efficient stimulators of naïve T cells. Namely, they massively upregulate HLA-DR expression on the cell surface, allowing to present *C. parvum*-derived peptides as novel antigens. This requires *de novo* expression of HLA-DR and loading of intracellularly stored molecules not yet loaded with peptides. Costimulatory molecules CD86 and especially CD40, in addition to CD80, are highly expressed, as primary activation of naïve T cells demands excellent costimulation that only mature DCs can provide. Adhesion molecules to stabilize the interaction with T cells are upregulated as well. MO-DCs express high levels of IL-6 and IL-8 upon *C. parvum*-interaction, necessary to shape T helper cell responses and to attract effector cells. MO-DCs efficiently take up *C. parvum* sporozoites and oocysts by phagocytosis and are highly mobile as required for migration to the T-cell areas of the draining lymph nodes. Equipped with many fine dendrites, the chance of interaction with naïve T cells detecting *C. parvum* peptides in context with fitting MHC molecules in the T-cell areas is markedly enhanced. No negative interference with the maturation process by *C. parvum* was observed.

We were prompted to study the effect of *C. parvum* on the maturation of DCs, considering the vulnerability of HIV patients to cryptosporidiosis, a direct hint that an efficient adaptive immune response against *C. parvum* is crucial for protection against this parasite. Bedi and Mead (40) presented some limited data that human DCs may respond to *C. parvum* antigens. The authors used recombinant *C. parvum* antigens and *C. parvum* sporozoites to stimulate human DCs. To demonstrate that their results were not caused by LPS contamination, especially as the authors purified their recombinant antigens from *Escherichia coli*, they used a Limulus (LAL) assay. The lower detection limit of this assay is, however, 0.03 Endotoxin Units (EU)/mL or, according to the definition of EU, 3–6 pg/mL LPS (41). A concentration of 2.5 pg/mL is, however, in our hands, already sufficient to induce maximal cytokine secretion by human DCs.

While not interfering with the induction of an adaptive immune response, *C. parvum* is nevertheless a highly successful parasite, given its worldwide prevalence and distribution. The parasite completes its life cycle within 4–5 days. It produces vast amounts of infective oocysts before adaptive immune responses are mounted to interfere with infection. As such, *C. parvum*-infected neonatal calves can shed up to 2 × 10^9^ oocysts during patency. Sequencing of the *C. parvum* genome (1) revealed that the relatively small genome size of only 9.1 Mb compared to larger genomes of *Plasmodium falciparum* (23 Mb) or other closely related apicomplexans (e.g., *Eimeria*, *Toxoplasma*, *Neospora*, and *Sarcocystis*) is “streamlined” for quick replication. Consequently, complete metabolic pathways like tricarboxylic acid cycle, oxidative phosphorylation, and *de novo* biosynthesis pathways for pyrimidines, amino acids, or cholesterol are lacking in contrast to other apicomplexans. Instead, *C. parvum* owns a large number of genes that encode for families of potential sugar and amino acid transporters, thereby indicating that it highly relies on the host cell metabolism for effective replication (1, 13, 42, 43).

Antigens of our *C. parvum* preparation stimulate the PRRs of DCs and induce maturation. It is not yet clear, however, if these antigens are originally expressed by *C. parvum* or by contaminating microorganisms. We have to keep in mind that *C. parvum* is surrounded by a plethora of commensal microorganisms in the gut, which carry many antigens that are readily detected by DCs via PRRs. As *C. parvum* cannot be cultured *in vitro*, at least not in sufficient numbers, our *C. parvum* preparations were purified from intestine/feces, being full of commensal microorganisms like LPS-containing gram-negative *E. coli.* Intestinal extracellular *C. parvum* stages (e.g., sporozoites, merozoites, gametocytes, and oocysts) may adhere to complete *E. coli*, LPS, or other microbial products either during purification *in vitro* or *in vivo* during infection of IECs in the gut. *C. parvum* crosses the sterile mucus layers and infects IECs. If they stick to microbial products, this would affect the immune response. When we analyzed the energy metabolism of DCs after treatment of MO-DCs with microorganisms and microbial products like flagellin (TLR-5 stimulus), LPS (TLR-4 stimulus), or complete yeast particles (TLR-2 stimulus due to zymosan), we found a rapid increase in ECAR, indicating that glycolysis was selectively enhanced to provide additional energy (**Supplementary Figure 6**), but not after treatment with *C. parvum* oocysts (**Figure 6**). After treatment with *C. parvum*, OCR was increased over time, indicating that OxPhos was enhanced. This difference may indicate that different PRRs may be addressed, especially taking into account that trace amounts of LPS already induce strong cytokine response in MO-DCs. Among other parasites of the group of apicomplexan, *Plasmodium* spp. are known to stimulate TLR-4 via glycosylphosphatidylinositols (GPIs), molecules also expressed in *C. parvum* (44–46). Given that *C. parvum*-derived responses entirely differed in kinetic and quality from LPS-mediated ones, theoretical contamination of *C. parvum* with LPS is less likely.

There is an indication from mice that *C. parvum* may induce TLR activation. *C. parvum*, however, does not infect immunocompetent wild-type mice or other rodents. Russler-Germain et al. discovered recently by chance that mice in their specific pathogen-free (SPF) murine facility were infected by a so far unknown strain of murine parasite *Cryptosporidium tyzzeri* (47), which escaped notice but was vertically transmitted in their facility and biased all T helper cell responses of infected animals. The complex host- and microbiome-dependent differences in *C. parvum*-related immunity studies evidence the need to extend scientific efforts beyond immunosuppressed murine models, which correctly reflect neither the *in vivo* small intestinal microbiome nor host immune reactions in the human system (13). Consequently, we here decided to avoid any mouse model and aimed instead to analyze human DC responses to the most prevalent human parasite to be as close as possible to the real parasite/host system.

We chose to generate primary human DCs from monocyte precursors for this study. Several intestinal DC populations were identified in the human gut, including plasmacytoid DCs, MO-DCs, and at least three DC1–DC2-type DCs, which favor Th-1 and Th-2 responses. The latter cells can be distinguished by differential expression of the markers CD103 and CD172 (SIRPα) (25). Despite their preference to induce certain T helper cell responses, pathogen-derived signals may override this decision. A Th-1 response is presumably protective against cryptosporidiosis. Tolerance induction against non-self antigens provided by food antigens and commensal microorganisms of the gut microbiome is another important matter. The lamina propria including the subepithelial dome-like structures of Peyer’s patches harbor plasmacytoid DCs and MO-DCs, and local inflammation, e.g., due to infection with *C. parvum*, will attract more plasmacytoid DCs and monocytes from the bloodstream. Part of the infiltrating monocytes will develop into MO-DCs and contribute to the immune response, and other MO-DCs will probably restore the depleted local intestinal DC pool (26). One advantage of choosing MO-DCs for this study is that they can be generated with ease from peripheral human blood in required numbers and purity. They are routinely removed from medical blood products generated from healthy blood donors, and such, buffy coats would be otherwise discarded, so they are the best available source. Resident DCs from the human intestine like DC1 cells would have been probably the obvious choice, but the use of this very limited material for this basic research purpose is questionable under strict ethical considerations. In our opinion, MO-DCs are, however, even the better choice. They are involved in the gut immune response as mentioned above (48), and most importantly, in contrast to resident DCs, they are most likely naïve for *C. parvum*, a prerequisite for this study. As mentioned before, *C. parvum* is ubiquitously present and infects constantly humans and livestock. Adult humans have for sure a history of infections, so resident DCs of the gut are most likely not naïve to *C. parvum* antigens. Typically, the intracellular development of *C. parvum* occurs within IECs. Nonetheless, in immuno-deficient humans, *C. parvum* may additionally spread to the biliary system, pancreatic duct, stomach, esophagus, and respiratory tract (49, 50). However, monocytes are derived from progenitors of the bone marrow, thus far away from the intestine, and released to the bloodstream with a circulating half-life of only 22 h (51). It is therefore unlikely that DCs, generated from such monocytes *in vitro*, were ever exposed to *C. parvum* by chance.

Still, the question remains why young children and neonatal calves are so vulnerable to severe life-threatening cryptosporidiosis. Given that children from third-world countries are mainly affected, malnutrition, heavy oocyst environmental contamination, and insufficient health care obviously play important roles in rendering this risk group so susceptible. The intestinal immune system, however, is unique, as it faces extraordinary challenges. After weaning, the mammalian intestine comes into contact with a plethora of novel but harmless food antigens. While we need to acquire tolerance against food antigens, we also need protection against food-borne pathogens. The GALTs change enormously in immune cell number and composition during the first years of life, thereby obviously improving current immune responses (52). Moreover, Kiner et al. (53) demonstrated that murine colon T helper cells do not follow the paradigm to differentiate into different stable T helper cell types but respond to the specific microbiome composition. Thus, changes in the gut microbiome during the first years of life significantly influence individual enteric immune responses. Consequently, the presence or absence of *Cryptosporidium* spp. will also contribute to these reactions. Overall, this study lays the foundation for a better understanding of immune response initiation in young/intestinal DCs.

## Data availability statement

The raw data supporting the conclusions of this article will be made available by the authors, without undue reservation.

## Ethics statement

All experiments and protocols were approved by the Ethics Committee of the Faculty of Medicine at the Justus Liebig University Giessen extended to Prof. Dr. Bein, Head of the Transfusion Medicine and Hemotherapy Centre (ZTH), UKGM, Giessen, Germany.

## Author contributions

RR: Conceptualization, Data curation, Methodology, Investigation, Writing – original draft, Writing – review & editing. FK: Conceptualization, Funding acquisition, Writing – review & editing. AK: Conceptualization, Writing – review & editing. AT: Conceptualization, Funding acquisition, Writing – review & editing. CH: Conceptualization, Funding acquisition, Writing – review & editing. SH: Data curation, Methodology, Investigation, Writing – original draft, Writing – review & editing. IC: Methodology, Investigation, Writing – review & editing. UG: Methodology, Investigation, Writing – review & editing.
