# Supplementary material for: Human dendritic cell interactions with the zoonotic parasite Cryptosporidium parvum result in activation and maturation
Source: Front Immunol. 2024 May 10;15:1388366. doi: 10.3389/fimmu.2024.1388366 (PMC11116633; doi:10.3389/fimmu.2024.1388366)
Supplement: Supplementary file 1 [file Presentation_1.pptx]

## Slide 1
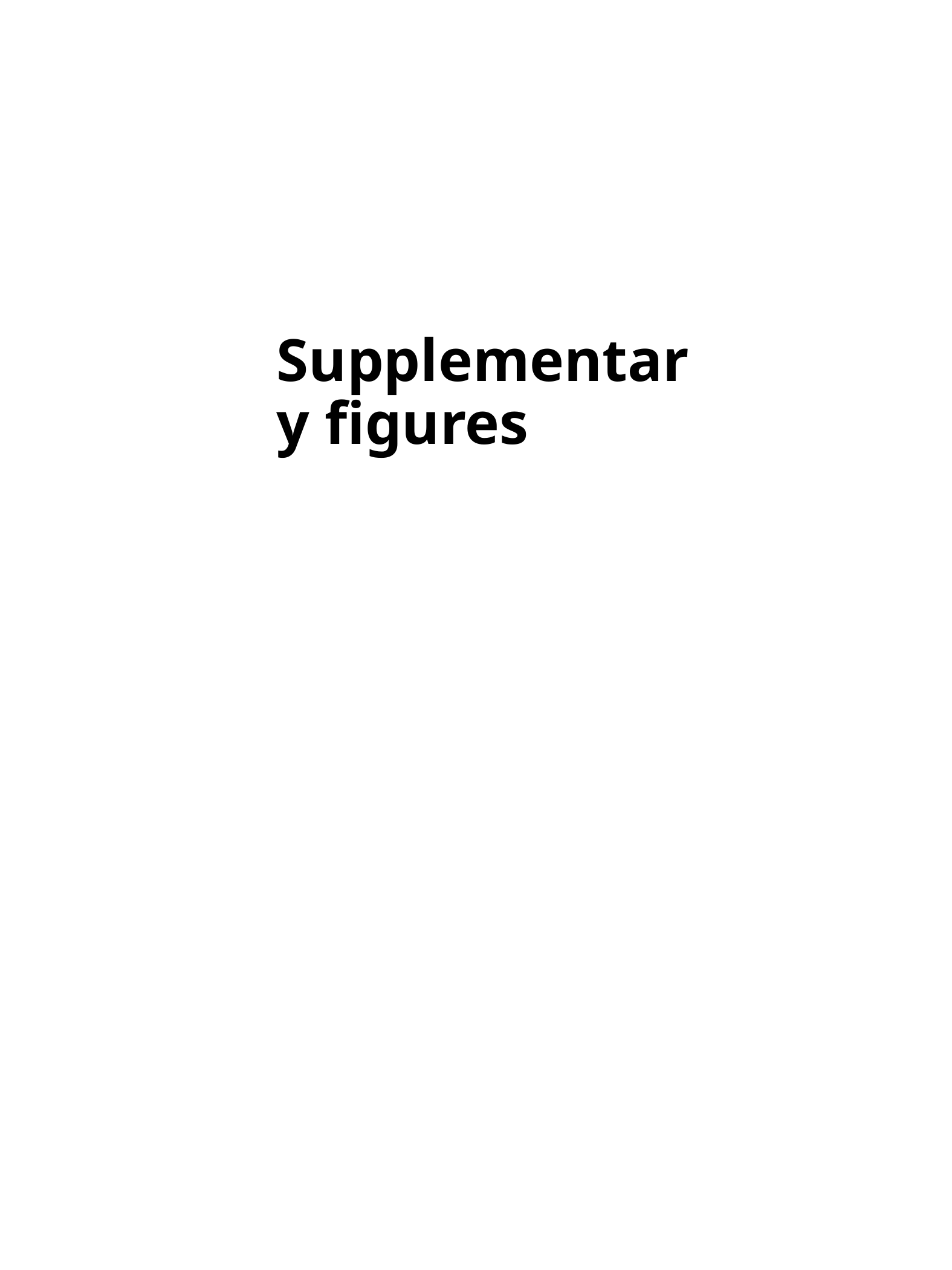

# Supplementary figures

## Slide 2
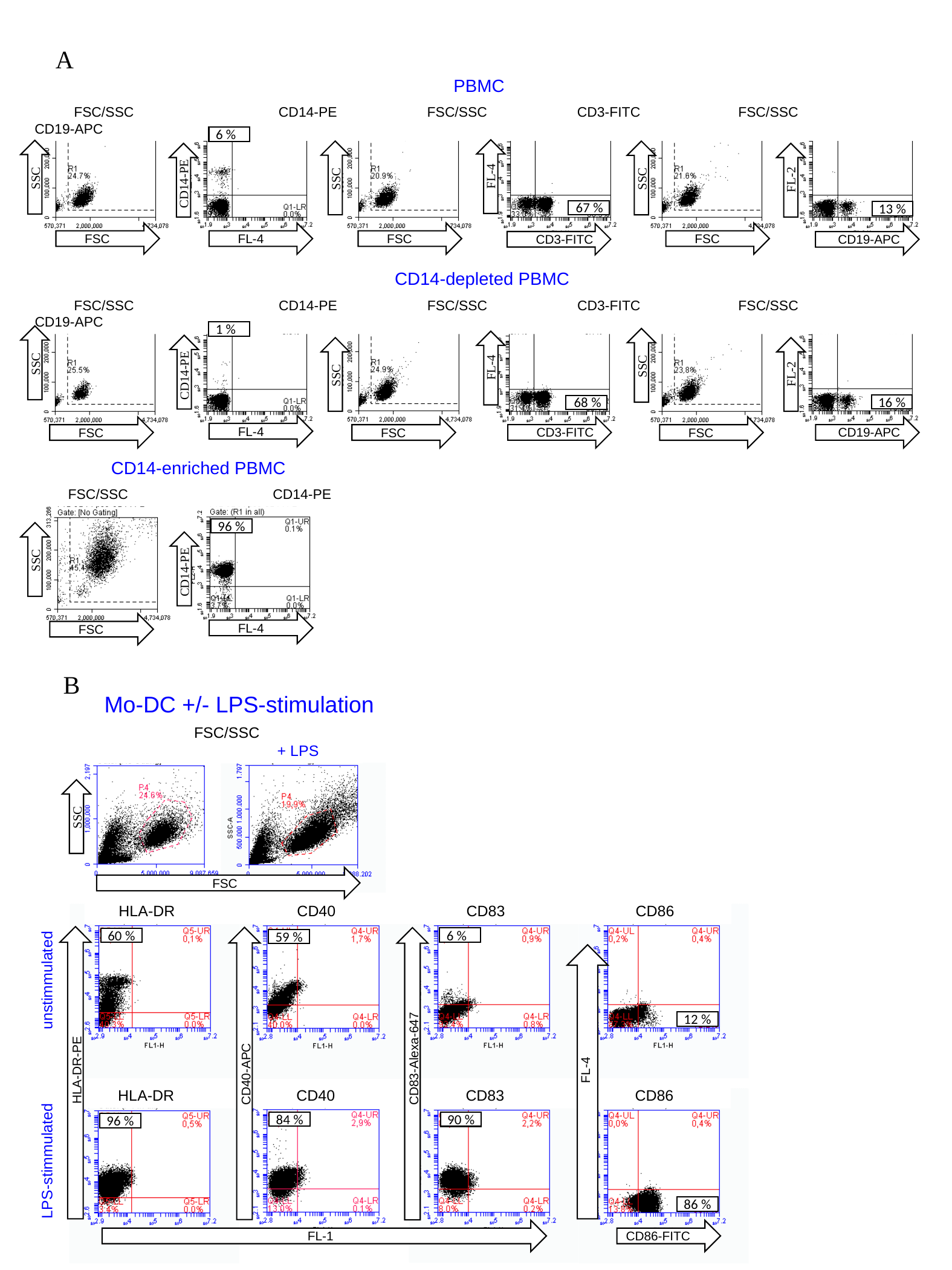

A
 PBMC
 FSC/SSC	 CD14-PE FSC/SSC CD3-FITC FSC/SSC CD19-APC
6 %
FL-4
SSC
SSC
SSC
CD14-PE
FL-2
FSC
FSC
FSC
FL-4
CD3-FITC
CD19-APC
67 %
13 %
CD14-depleted PBMC
 FSC/SSC	 CD14-PE FSC/SSC CD3-FITC FSC/SSC CD19-APC
1 %
SSC
SSC
FL-4
CD14-PE
SSC
FL-2
FL-4
CD3-FITC
FSC
FSC
FSC
CD19-APC
68 %
16 %
CD14-enriched PBMC
 FSC/SSC	 CD14-PE
96 %
SSC
CD14-PE
FL-4
FSC
B
Mo-DC +/- LPS-stimulation
FSC/SSC
 + LPS
FSC
SSC
FL-4
HLA-DR-PE
CD83-Alexa-647
CD40-APC
 HLA-DR CD40 CD83 CD86
6 %
60 %
59 %
 unstimmulated
 FL-1
12 %
 HLA-DR CD40 CD83 CD86
84 %
90 %
96 %
LPS-stimmulated
CD86-FITC
86 %

## Slide 3
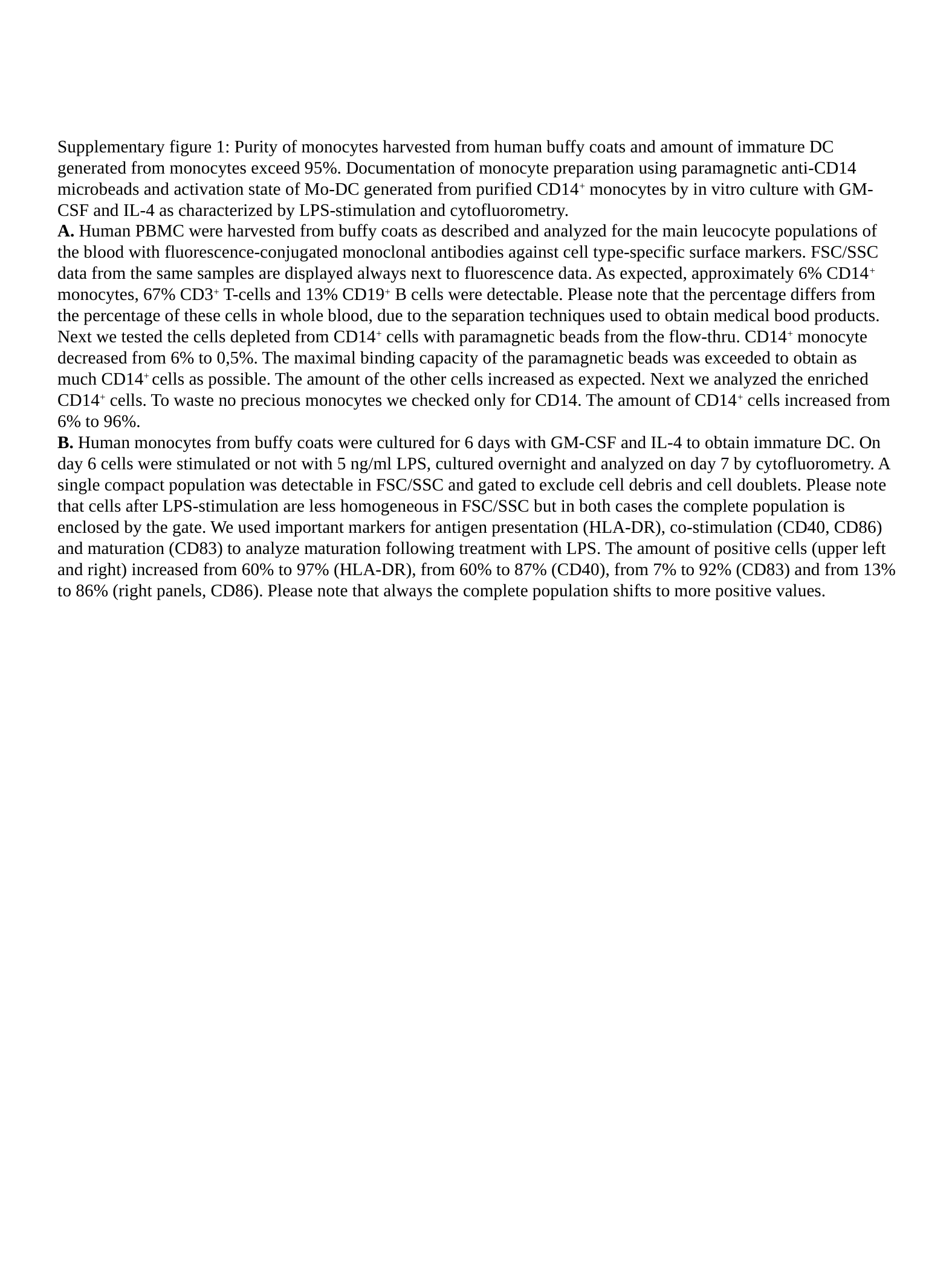

Supplementary figure 1: Purity of monocytes harvested from human buffy coats and amount of immature DC generated from monocytes exceed 95%. Documentation of monocyte preparation using paramagnetic anti-CD14 microbeads and activation state of Mo-DC generated from purified CD14+ monocytes by in vitro culture with GM-CSF and IL-4 as characterized by LPS-stimulation and cytofluorometry.
A. Human PBMC were harvested from buffy coats as described and analyzed for the main leucocyte populations of the blood with fluorescence-conjugated monoclonal antibodies against cell type-specific surface markers. FSC/SSC data from the same samples are displayed always next to fluorescence data. As expected, approximately 6% CD14+ monocytes, 67% CD3+ T-cells and 13% CD19+ B cells were detectable. Please note that the percentage differs from the percentage of these cells in whole blood, due to the separation techniques used to obtain medical bood products. Next we tested the cells depleted from CD14+ cells with paramagnetic beads from the flow-thru. CD14+ monocyte decreased from 6% to 0,5%. The maximal binding capacity of the paramagnetic beads was exceeded to obtain as much CD14+ cells as possible. The amount of the other cells increased as expected. Next we analyzed the enriched CD14+ cells. To waste no precious monocytes we checked only for CD14. The amount of CD14+ cells increased from 6% to 96%.
B. Human monocytes from buffy coats were cultured for 6 days with GM-CSF and IL-4 to obtain immature DC. On day 6 cells were stimulated or not with 5 ng/ml LPS, cultured overnight and analyzed on day 7 by cytofluorometry. A single compact population was detectable in FSC/SSC and gated to exclude cell debris and cell doublets. Please note that cells after LPS-stimulation are less homogeneous in FSC/SSC but in both cases the complete population is enclosed by the gate. We used important markers for antigen presentation (HLA-DR), co-stimulation (CD40, CD86) and maturation (CD83) to analyze maturation following treatment with LPS. The amount of positive cells (upper left and right) increased from 60% to 97% (HLA-DR), from 60% to 87% (CD40), from 7% to 92% (CD83) and from 13% to 86% (right panels, CD86). Please note that always the complete population shifts to more positive values.

## Slide 4
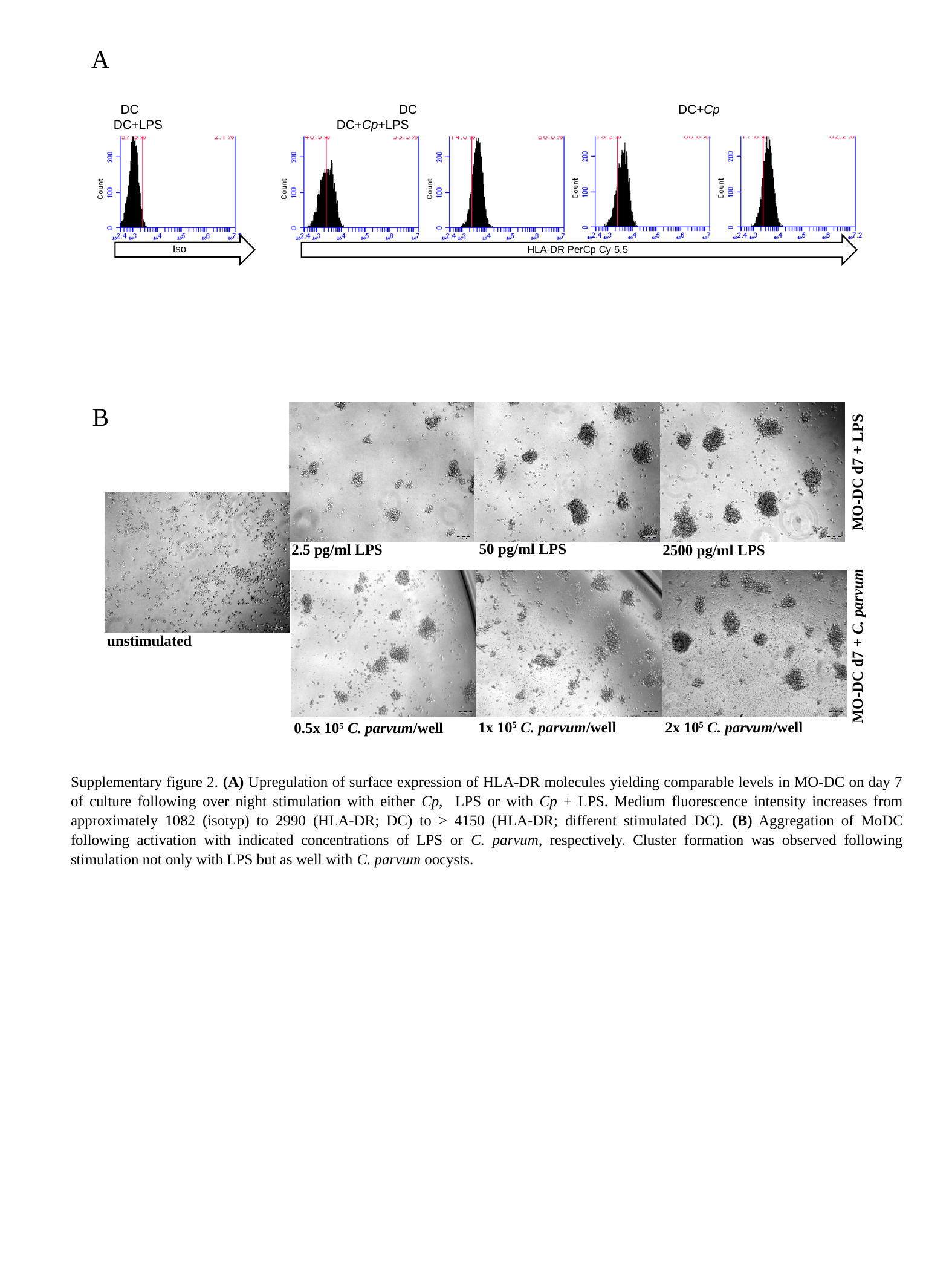

A
 DC 		 DC 			 DC+Cp 		 DC+LPS 		 DC+Cp+LPS
Iso
HLA-DR PerCp Cy 5.5
B
MO-DC d7 + LPS
50 pg/ml LPS
2.5 pg/ml LPS
2500 pg/ml LPS
MO-DC d7 + C. parvum
unstimulated
2x 10^5^ C. parvum/well
1x 10^5^ C. parvum/well
0.5x 10^5^ C. parvum/well
Supplementary figure 2. (A) Upregulation of surface expression of HLA-DR molecules yielding comparable levels in MO-DC on day 7 of culture following over night stimulation with either Cp, LPS or with Cp + LPS. Medium fluorescence intensity increases from approximately 1082 (isotyp) to 2990 (HLA-DR; DC) to > 4150 (HLA-DR; different stimulated DC). (B) Aggregation of MoDC following activation with indicated concentrations of LPS or C. parvum, respectively. Cluster formation was observed following stimulation not only with LPS but as well with C. parvum oocysts.

## Slide 5
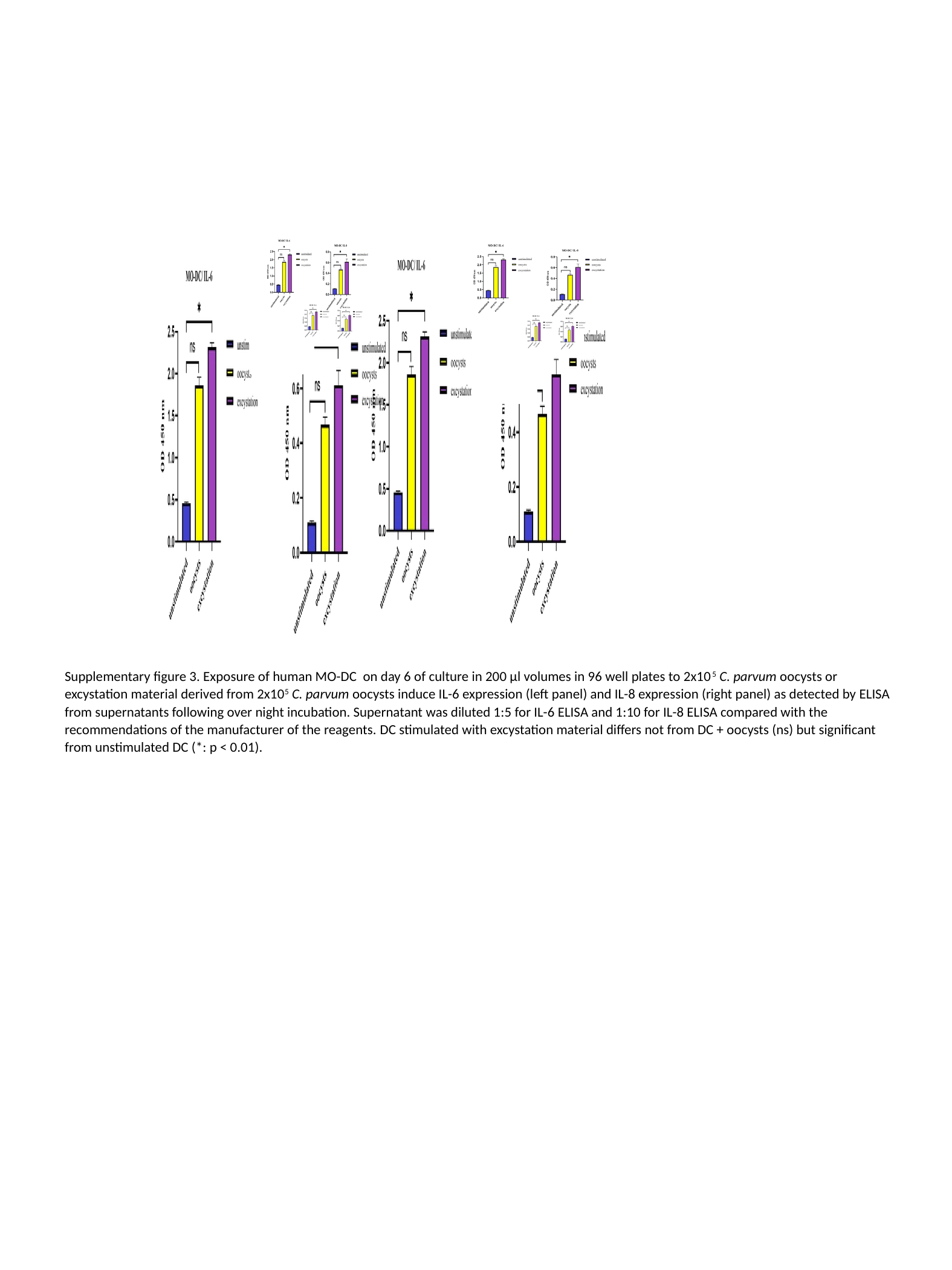

Supplementary figure 3. Exposure of human MO-DC on day 6 of culture in 200 µl volumes in 96 well plates to 2x10^5^ C. parvum oocysts or excystation material derived from 2x10^5^ C. parvum oocysts induce IL-6 expression (left panel) and IL-8 expression (right panel) as detected by ELISA from supernatants following over night incubation. Supernatant was diluted 1:5 for IL-6 ELISA and 1:10 for IL-8 ELISA compared with the recommendations of the manufacturer of the reagents. DC stimulated with excystation material differs not from DC + oocysts (ns) but significant from unstimulated DC (*: p < 0.01).

## Slide 6
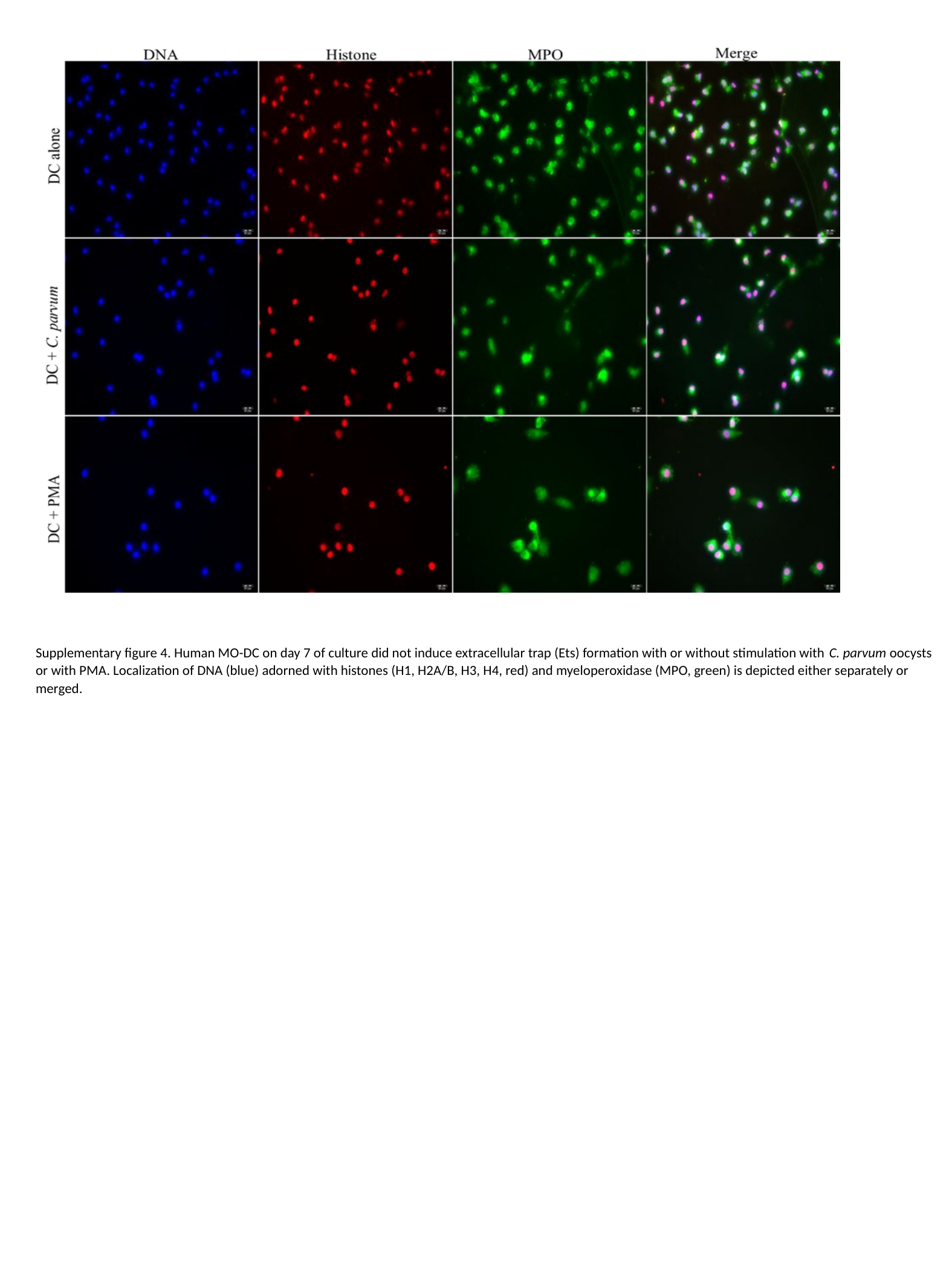

Supplementary figure 4. Human MO-DC on day 7 of culture did not induce extracellular trap (Ets) formation with or without stimulation with C. parvum oocysts or with PMA. Localization of DNA (blue) adorned with histones (H1, H2A/B, H3, H4, red) and myeloperoxidase (MPO, green) is depicted either separately or merged.

## Slide 7
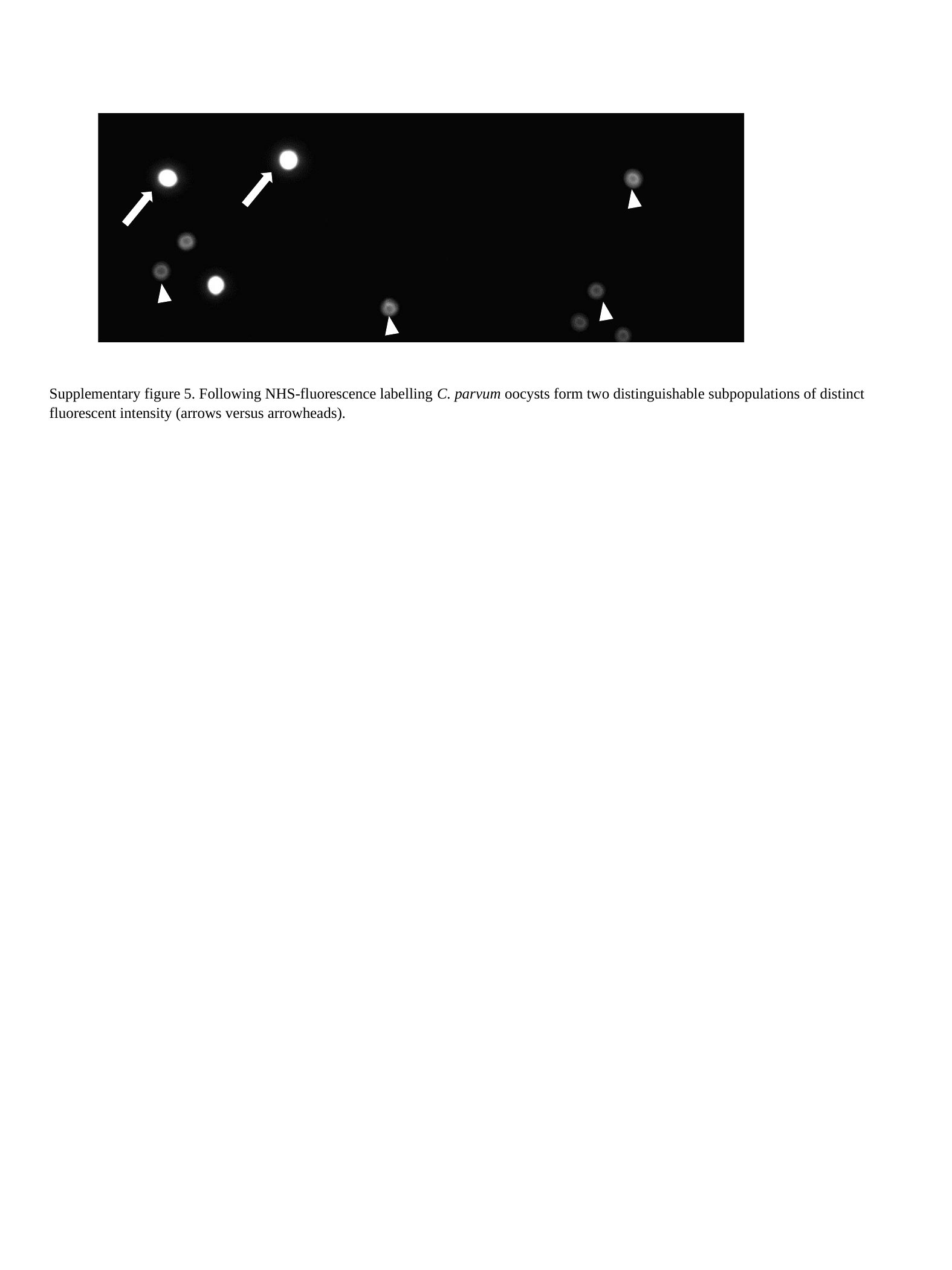

Figure
Supplementary figure 5. Following NHS-fluorescence labelling C. parvum oocysts form two distinguishable subpopulations of distinct fluorescent intensity (arrows versus arrowheads).

## Slide 8
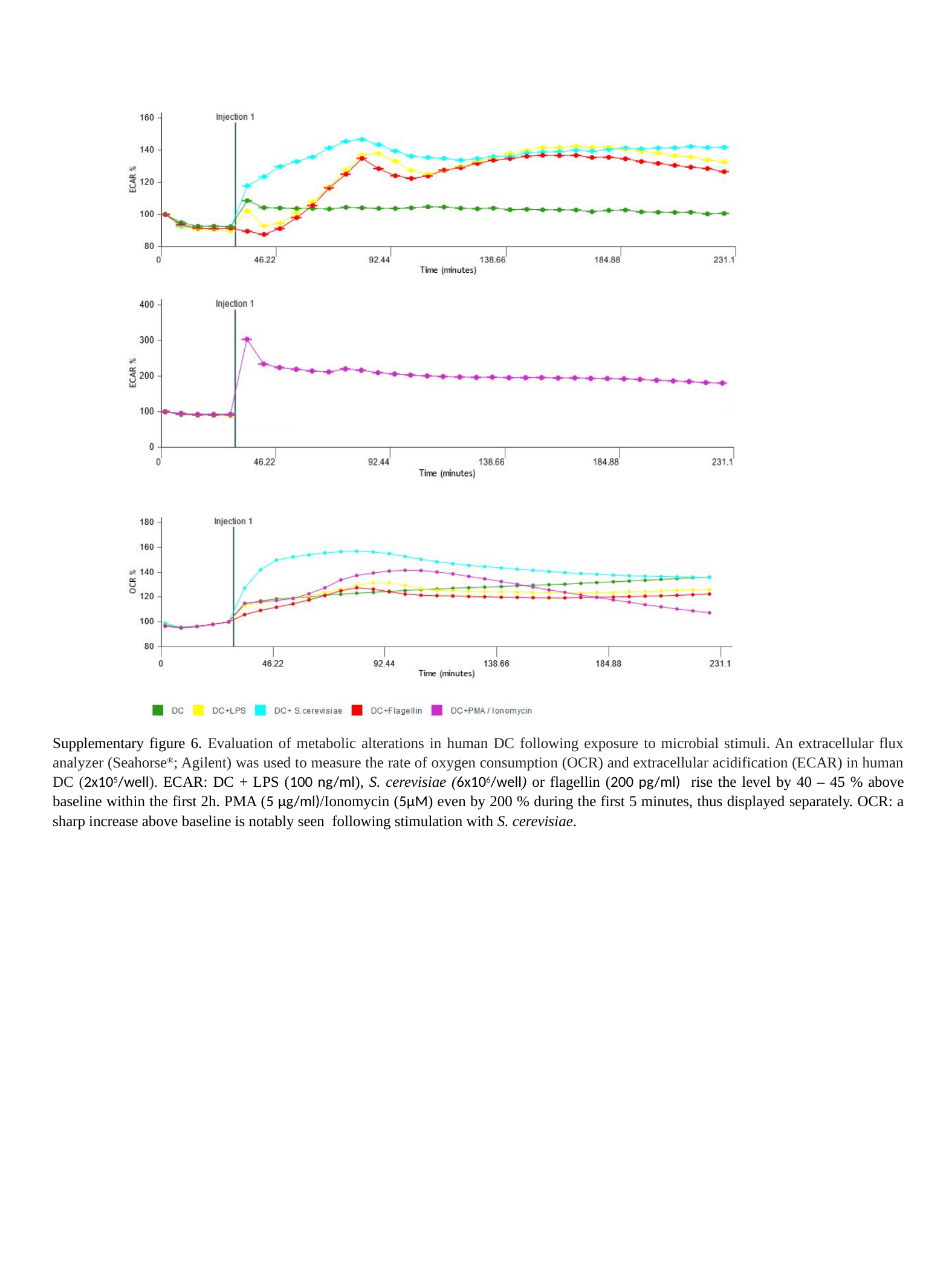

Supplementary figure 6. Evaluation of metabolic alterations in human DC following exposure to microbial stimuli. An extracellular flux analyzer (Seahorse®; Agilent) was used to measure the rate of oxygen consumption (OCR) and extracellular acidification (ECAR) in human DC (2x10^5^/well). ECAR: DC + LPS (100 ng/ml), S. cerevisiae (6x10^6^/well) or flagellin (200 pg/ml) rise the level by 40 – 45 % above baseline within the first 2h. PMA (5 µg/ml)/Ionomycin (5µM) even by 200 % during the first 5 minutes, thus displayed separately. OCR: a sharp increase above baseline is notably seen following stimulation with S. cerevisiae.
